# Flow-based imaging reveals dissociated monocyte adhesion and transmigration patterns between TNF-α and diabetes-induced vascular inflammation

**DOI:** 10.3389/fimmu.2026.1827607

**Published:** 2026-07-10

**Authors:** Dilvin Semo, Xiaohe Liang, Mariel Schwietzer, Weiqi Wang, Xiaokang Hu, Marc Dorenkamp, Prabhakaran Poorana Priya, Joshua Martin Manogaran, Franklin Christopher Vincent, Holger Reinecke, Rinesh Godfrey

**Affiliations:** 1 Vascular Signaling, Molecular Cardiology, Department of Cardiology I - Coronary and Peripheral Vascular Disease, Heart Failure, University Hospital Münster, Münster, Germany; 2 Department of Cardiology I- Coronary and Peripheral Vascular Disease, Heart Failure, University Hospital Münster, Münster, Germany; 3 Department of Pathology, Velammal Medical College Hospital and Research Institute, Madurai, India; 4 Acute Medicine, Hillingdon Hospital, London, United Kingdom; 5 Department of Biology, University of Maryland Global Campus Europe (UMGC-Europe), Kaiserslautern, Germany

**Keywords:** adhesion, diapedesis, flow-based imaging, immune-metabolic dysfunction, monocytes, reverse transmigration, T2DM, vascular inflammation

## Abstract

Monocyte–endothelial interactions drive both acute inflammation and chronic vascular disease in type 2 diabetes mellitus (T2DM), yet whether TNF-α–induced and diabetes-associated monocyte trafficking engage similar or distinct molecular programs remains unclear. Using a physiological flow-based imaging system, we quantified CD14^+^ monocyte adhesion, transendothelial migration (TEM), and abluminal residence on HUVEC monolayers activated with TNF-α or T2DM serum under controlled shear stress. Both stimuli induced comparable monocyte adhesion (~40 cells by 3 min), but TEM efficiency diverged markedly: TNF-α promoted robust TEM (~18% by 5 min) whereas T2DM conditions showed severely impaired TEM (~5%). Under T2DM conditions, monocytes exhibited prolonged abluminal retention (median 85 min vs. 25 min; p<0.0001), a phenotype recapitulated by endothelial hyperglycemic exposure alone. Comparative RNA-seq analysis of T2DM (GSE92724) and TNF-α–stimulated (GSE134489) endothelial cells revealed near-zero transcriptional correlation (r = 0.018) between conditions. TNF-α drove coordinate NF-κB–dependent upregulation of VCAM1, ICAM1, E-selectin, and junctional molecules with organized junctional remodeling, whereas T2DM produced VCAM1-biased adhesion with ICAM1 downregulation (0.61-fold), claudin suppression, and junctional disorganization. KEGG pathway mapping confirmed organized endothelial–leukocyte integrin co-activation under TNF-α versus discoordination junctional loss under T2DM. qPCR validation identified selective RAGE and JAM3 upregulation under T2DM—consistent with AGE-RAGE signaling and JAM-3–MAC-1 monocyte trapping—without classical NF-κB activation. These findings define two fundamentally distinct paradigms: a TNF-α “recruitment model” enabling efficient trafficking and resolution, versus a T2DM “retention model” characterized by VCAM1-biased adhesion, junctional disorganization, and impaired reverse transmigration driving chronic monocyte accumulation, identifying AGE-RAGE signaling and JAM-3–MAC-1 interactions as potential therapeutic targets for diabetic vascular inflammation.

## 1 Introduction

Type 2 diabetes mellitus (T2DM) represents one of the most significant global health challenges of the 21st century. According to the International Diabetes Federation (IDF), approximately 537 million adults were living with diabetes worldwide in 2021, a figure projected to rise to 783 million by 2045, with T2DM accounting for over 90% of cases (1). Diabetes is the leading cause of preventable blindness, end-stage renal disease, and non-traumatic lower limb amputation, and it confers a two- to four-fold increased risk of cardiovascular disease (CVD), which remains the primary cause of mortality in people with T2DM, responsible for approximately 50% of all diabetes-related deaths (2, 3). The annual global economic burden attributable to diabetes exceeds USD 966 billion in health expenditure, underscoring the urgent need to understand the pathogenic mechanisms linking metabolic dysregulation to vascular complications (1).

Excessive monocyte accumulation in atherosclerotic lesions exacerbates vascular complications in type 2 diabetes mellitus (T2DM), yet the mechanisms underlying diabetic monocyte–endothelial dysfunction remain inadequately elucidated (4–6). Monocytes control inflammation by adhering to the endothelium, migrating through it (TEM), and then infiltrating tissues, where they can become macrophages or dendritic cells (7, 8). Inflammatory signals and metabolic factors tightly control these trafficking processes. When they go wrong in diabetes, they speed up atherosclerosis and heart problems (6, 9). Recent evidence suggests that monocyte accumulation in diabetic vessel walls may not primarily result from enhanced recruitment, but rather from impaired reverse transendothelial migration (rTEM) and prolonged abluminal retention (10). We recently showed that T2DM conditions severely impair the rTEM capacity of CD14^+^CD16^-^ monocytes through enhanced junctional adhesion molecule-3 (JAM-3)–macrophage-1 antigen (MAC-1) integrin interactions, establishing a retention-based mechanism that shifts therapeutic targeting from recruitment blockade toward restoration of monocyte egress (10). This discovery corresponds with the foundational finding by Bradfield et al. that JAM-C at endothelial junctions serves as a gatekeeper for unidirectional monocyte migration, and that the disruption of JAM-B/JAM-C interactions enhances reverse transmigration instead of diminishing forward transendothelial migration (TEM) (11).

TNF-α plays a multifaceted and cell-autonomous role in monocyte biology that extends beyond its function as an endothelial activator. *In vivo*, monocyte-intrinsic TNF signaling through TNFR1 and TNFR2 is required for monocyte survival, maintenance, and efficient egress from the bone marrow; monocytes deficient in TNF or its receptors show impaired competitive fitness and reduced infiltration of Ly6C^hi^ effector monocytes at sites of inflammation (12). The clinical significance of this axis is underscored by chronic inflammatory diseases in which dysregulated TNF-driven monocyte–endothelial interactions play a central pathogenic role. In Crohn’s disease (CD), TNF-α produced by activated lamina propria macrophages accumulates in close proximity to mucosal micro vessels and disrupts the vascular endothelium (13), driving upregulation of ICAM-1, VCAM-1, and E-selectin and facilitating excessive monocyte recruitment to inflamed sites (14, 15). Monocyte-derived macrophages recruited from peripheral blood are a histological hallmark of CD lesions (16), and anti-TNF biologic agents such as infliximab reduce endothelial adhesion molecule expression, induce apoptosis of activated monocytes, and improve vascular dysfunction in CD and rheumatoid arthritis (17, 18). Notably, in a TNF-overproduction mouse model of Crohn’s-like ileitis, systemic TNF excess expanded circulating monocyte numbers and promoted a shift toward Ly6C^-^ non-classical monocytes *in vivo*, while B cell-derived LTα3 — acting through the same TNFR1/TNFR2 receptors — unexpectedly counterbalanced TNF-driven pathology, highlighting the inherent complexity of the *in vivo* TNF milieu and reinforcing the value of defined recombinant TNF as a mechanistically tractable benchmark in controlled *in vitro* systems (19). These observations collectively establish TNF-α as a master regulator of monocyte–endothelial interactions *in vivo*, spanning both steady-state trafficking and disease-associated inflammation.

The endothelium modulates monocyte trafficking via the coordinated expression of adhesion molecules and junctional proteins in response to inflammatory and metabolic stimuli. Acute inflammatory stimuli, such as TNF-α, rapidly upregulate ICAM-1 and VCAM-1 via NF-κB and protein kinase C–dependent pathways, facilitating robust monocyte adhesion and transmigration (20). The multi-step adhesion cascade—now understood to encompass selectin-mediated rolling, chemokine-triggered activation, integrin-dependent arrest, intraluminal crawling, and paracellular or transcellular migration (21, 22)—proceeds efficiently under TNF-α activation, with E-selectin and P-selectin mediating the initial rolling interactions (23–25). Conversely, the chronic diabetic environment—characterized by hyperglycemia, advanced glycation end-products (AGEs), oxidative stress, and low-grade inflammation—stimulates endothelial cells via distinct pathways that may yield qualitatively different monocyte–endothelial interactions (26). AGEs cause damage to blood vessels in people with diabetes by binding to the receptor for advanced glycation end-products (RAGE). This activates NF-κB, causes oxidative stress, and increases the levels of adhesion molecules like VCAM-1 (27, 28). Hyperglycemia has been demonstrated to augment monocyte–endothelial adhesion via endothelial IL-8 production and connexin-43 overexpression (26, 29). However, it is still not clear whether the diabetic environment uses molecular mechanisms that are similar to TNF-α or completely different transcriptional programs. It is also unclear how these differences affect the kinetics of adhesion-to-transmigration and the fate of cells after they have migrated.

Although comprehending these mechanistic differences is clinically significant, the majority of current *in vitro* adhesion assays utilize static culture conditions that fail to replicate physiological hemodynamic forces. Shear stress alters the expression of adhesion molecules, with research indicating that flow conditions elevate ICAM-1 expression while diminishing VCAM-1 and E-selectin expression in TNF-α–stimulated endothelium (30). Quantifying monocyte dynamics in real-time under physiologically relevant flow conditions remains technically difficult. Flow-based imaging systems mitigate this limitation by facilitating the concurrent visualization of rolling, firm adhesion, and transmigration under regulated shear stress (31); however, such assays have yet to be systematically employed to directly compare inflammatory and metabolic-inflammatory endothelial activation models, nor have they been amalgamated with transcriptomic profiling to elucidate the upstream molecular programs that differentiate these conditions.

In this study, we created a physiological flow-based imaging system to measure CD14^+^ monocyte-endothelial interactions in controlled conditions that mimic postcapillary venules. We also compared TNF-α-mediated endothelial activation with the metabolically driven, low-grade inflammatory state associated with T2DM. We utilized a dual-model methodology: TNF-α–activated endothelium was selected as a defined, mechanistically well-characterized reference condition representing classical cytokine-driven acute endothelial activation, rather than as a surrogate for any specific TNF-driven clinical entity; T2DM patient serum-treated endothelium represents the complex metabolic-inflammatory milieu encountered in the diabetic vasculature. This experimental design allows the well-characterized NF-κB–dependent endothelial response to TNF-α to serve as a canonical comparator against which the transcriptional and functional divergence of the diabetic condition can be systematically defined. We used monocytes from both healthy donors and T2DM patients that were isolated by negative selection to ensure high purity and preserve functional integrity (32). We wanted to achieve three specific goals (1): to find out if TNF-α and T2DM conditions have similar or different effects on monocyte adhesion, transmigration kinetics, and post-transmigration behavior; (2) to find out if T2DM conditions cause monocyte accumulation through recruitment-based or retention-based mechanism; and (3) identify the transcriptional programs that distinguish TNF-α from T2DM endothelial activation using RNA-seq data, linking molecular signatures to the functional dissociation between adhesion and transmigration. We posited that the diabetic environment elicits mechanistically distinct effects in contrast to acute inflammatory activation, as evidenced at both transcriptional and functional levels, which can be elucidated through single-cell imaging under physiologically relevant flow conditions, complemented by pathway-level transcriptomic analysis.

## 2 Materials and methods

### 2.1 Monocyte and human plasma collection from clinical cohorts and healthy individuals

CD14+CD16- human monocytes were isolated from healthy donors and from non-T2DM individuals or T2DM patients according to published protocols (32, 33) using magnetic-activated cell sorting (MACS) negative selection with the Monocyte Isolation Kit II (Miltenyi Biotec). Peripheral blood was collected into EDTA-coated tubes and processed within 4 hours. Peripheral blood mononuclear cells (PBMCs) were isolated by Histopaque-1077 density-gradient centrifugation, washed with PBS, and optionally pre-enriched using a discontinuous Percoll gradient. Cell purity (>95%) and viability (>98%) were confirmed by flow cytometry (CD14-FITC, CD16-PE, propidium iodide).

### 2.2 Primary cells used for experiments

Human umbilical vein endothelial cells (HUVECs) were maintained at low passage numbers (passages 1–3) and cryopreserved in multiple batches at identical passage numbers to minimize phenotypic drift and reduce experimental variability. Classical CD14^+^ monocytes were isolated from healthy donor peripheral blood by density-gradient centrifugation followed by MACS and cryopreserved in liquid nitrogen prior to experimental use to provide scheduling flexibility while maintaining functional integrity for downstream flow-based adhesion assays.

### 2.3 RNA isolation and qPCR

Total RNA was extracted from 5–8 × 10^6^ monocytes using the NucleoSpin RNAKit (Macherey-Nagel). For *in vitro* experiments, RNA was isolated 8–12 h after treatment, and cDNA was synthesized with the RevertAid First Strand cDNA Synthesis Kit (Thermo Scientific). qPCR was performed using iTaq™ Universal SYBR Green Supermix (Bio-Rad), and relative mRNA expression was calculated by the 2^-^ΔΔCt method with YWHAZ as the reference gene. Primer sequences are listed in **Supplementary Table 1**.

### 2.4 Bioinformatic analysis of publicly available RNA-seq datasets

Publicly available bulk RNA-seq datasets were obtained from the NCBI Gene Expression Omnibus (GEO). For T2DM endothelial transcriptomics, dataset GSE92724 was used, comprising FACS-sorted (CD31^+^CD45^-^Podoplanin^-^) dermal endothelial cells from T2DM patients (n=4) and normoglycemic controls (n=6) (34). For TNF-α–stimulated endothelial transcriptomics, dataset GSE134489 was used, comprising human coronary artery endothelial cells (HCAECs) treated with TNF-α (20 ng/mL, 18 h; n=3) or vehicle control (n=3); only control siRNA-treated samples (GSM3953336–GSM3953341) were included to exclude confounding effects of ACSL3 knockdown (35). Raw count matrices were downloaded and processed in R (version 4.5.1; R Core Team, 2025). Low-expressed genes were filtered by requiring a minimum count threshold across samples, and differential expression analysis was performed using DESeq2 with default parameters, including library size normalization via median-of-ratios and Wald test statistics (36). Genes were considered significantly differentially expressed at an adjusted P-value < 0.05 (Benjamini–Hochberg correction) and |log_2_ fold change| > 1. Principal component analysis (PCA) was performed on variance-stabilizing transformed (VST) counts to assess sample clustering and identify outliers. Volcano plots were generated using the EnhancedVolcano R package (37), and heatmaps of selected adhesion and junctional genes were generated using the heatmap package with z-score–normalized expression values (38). Pearson correlation analysis of log_2_ fold changes between the T2DM and TNF-α datasets was performed to assess the degree of transcriptional overlap. For pathway-level visualization, differentially expressed genes were mapped onto the KEGG Leukocyte transendothelial migration pathway (hsa04670) using the pathview R package, with log_2_ fold-change values overlaid as color gradients for both conditions independently (39). Gene Ontology (GO; biological process, cellular component, and molecular function) and KEGG pathway enrichment analyses were conducted using the clusterProfiler R package (v4.16.0) with org.Hs.eg.db for gene identifier mapping (40). Enrichment significance was assessed using a Benjamini–Hochberg-adjusted p-value threshold of <0.05. All bioinformatic analyses were performed in R (v4.5.1) using Bioconductor packages.

### 2.5 FACS staining and analysis

1.5–2.0 × 10^6^ monocytes were washed once with ice-cold PBS, fixed with 80% methanol, and stained in FACS buffer (PBS with 1% BSA and 0.1% sodium azide) for 30 min at 4 °C. After staining, cells were washed with PBS and analysis was performed using Guava easyCyte (Millipore). The following antibodies from Miltenyi Biotec were used: CD14-FITC (clone TÜK4, 130-098-058), CD62L-PE (clone 145/15), CD162-FITC (clone REA319), CD106 (VECAM-1)-PE (130-129-507), CD54 (ICAM-1)-APC (130-121-342), CD62E (E-Selectin)-APC (130-104-644.

### 2.6 HUVEC expansion and maintenance

Cryopreserved passage-1 HUVECs were cultivated as described before (41). Cells were rapidly thawed in a 37 °C water bath, resuspended in complete M199 [M199, 10% FBS, 1% penicillin/streptomycin, bovine pituitary extract, heparin], and seeded into 75 cm² tissue-culture flasks coated with 1% gelatin for 30 minutes at 37 °C (41). Cultures were incubated at 37 °C/5% CO_2_ with medium changes every 48 hours. At 80–90% confluence, cells were detached with Accutase for ≤5 minutes and reseeded at 5,000 cells/cm² or cryopreserved at 0.5–1.0 × 10^6^ cells/mL in 90% FBS/10% DMSO using a −1 °C/min controlled-rate freezer before transfer to liquid nitrogen. All experiments utilized passages 2–3 to minimize phenotypic drift.

### 2.7 Endothelial monolayer preparation

Ibidi μ-Slide VI channels were precoated with 0.1% gelatin for 30 minutes, rinsed, and allowed to dry. HUVECs were seeded at 2.0 × 10^5^ cells/channel in endothelial growth medium (M199 supplemented with 10% FBS, 1% penicillin/streptomycin, and 30 µg/mL heparin) and cultured for 24–48 hours until a uniform, gap-free monolayer formed, as verified by phase-contrast microscopy. Only slides exhibiting complete confluence were advanced to flow assays.

### 2.8 Pro-inflammatory activation

Confluent monolayers were stimulated with 10 ng/mL recombinant human TNF-α in fresh endothelial medium for 4–6 hours at 37 °C/5% CO_2_ to induce surface expression of ICAM-1 and VCAM-1. Following activation, slides were gently washed with pre-warmed medium immediately before introduction into the flow chamber system. For all TNF-α experiments, the control condition consisted of HUVEC monolayers incubated in matched fresh endothelial medium for the same duration without cytokine; TNF-α was reconstituted in sterile PBS and added at a volume representing ≤0.1% of total medium volume, rendering a separate vehicle-only arm functionally indistinguishable from the medium control.

### 2.9 T2DM patient serum collection and endothelial treatment

Venous blood from T2DM patients (n=6) and age-matched non-diabetic controls (n=6) was collected into serum separator tubes, allowed to clot for 30 min at room temperature, and centrifuged at 2,000 × g for 10 min at 4 °C. Serum was aliquoted and stored at −80 °C until use. For endothelial activation, T2DM patient serum was added to confluent HUVEC monolayers at 20% v/v in M199 culture medium for 6–24 hours at 37 °C/5% CO_2_. For serum experiments, the control condition consisted of HUVEC monolayers treated with 20% (v/v) serum from age-matched non-diabetic donors (n=6) for the same duration and under identical culture conditions as T2DM serum treatment. This donor-matched design ensures that any observed differences in monocyte–endothelial interactions reflect the diabetic serum milieu specifically, rather than non-specific effects of serum supplementation.

### 2.10 Flow assay

Cryopreserved CD14^+^ monocytes were thawed rapidly in a 37 °C water bath, resuspended at ≤2.5 × 10^6^ cells/mL in complete RPMI-1640, and incubated for ≥6 hours at 37 °C/5% CO_2_ to restore metabolic activity before perfusion. An inverted microscope (ZEISS AxioObserver) was configured for time-lapse acquisition every 15 seconds at 10× magnification for 2 hours. Silicone tubing (0.8 mm inner diameter) was flushed with PBS and pre-warmed medium to remove air, then connected to a syringe pump adjusted to 0.12–0.20 mL/min to reproduce post-capillary venule shear rates. Wall shear stress (τ) was calculated using the validated analytical formula for the Ibidi µ-Slide VI 0.4 channel geometry: τ = η × 176.1 × Φ, where τ is wall shear stress (dyn/cm²), η is the dynamic viscosity of aqueous medium at 37 °C (~0.0072 dyn·s/cm²), and Φ is the volumetric flow rate (mL/min), as provided in ibidi Application Note 11 (ibidi GmbH, Gräfelfing, Germany). At a pump setting of 0.12–0.20 mL/min, this yields τ ≈ 0.15–0.25 dyn/cm² (0.015–0.025 Pa), corresponding to low venular shear conditions consistent with post-capillary venule hemodynamics *in vivo*. The same flow range was used in the founding characterization of the monocyte TEM flow assay employed here, where a shear stress of 0.05 Pa was described as representative of small venule shear rates (11) and was employed and accepted through peer review in our previous study using the identical Ibidi µ-Slide VI setup (10). Ibidi μ-Slide VI chambers bearing activated HUVEC monolayers were mounted in a stage-top incubator maintained at 37 °C/5% CO_2_. Immediately before perfusion, 1.5 × 10^6^ monocytes were resuspended in 300 µL of 37 °C RPMI-1640 and introduced into the flow circuit through a sterile syringe, ensuring bubble-free delivery. Time-lapse recording commenced synchronously with perfusion, capturing monocyte–endothelial interactions at 15-second intervals for 2 hours. Prior to flow assays, monocyte viability and purity were confirmed by flow cytometry using propidium iodide exclusion and CD14 staining, respectively. Only preparations with viability >98% and CD14^+^ purity >95% were used. A minimum 6-hour recovery incubation at 37 °C/5% CO_2_ in RPMI supplemented with 10% autologous plasma was performed after thawing to restore membrane integrity and baseline migratory capacity before perfusion.

### 2.11 Analysis of cellular interactions

Time-lapse sequences were analyzed using Fiji ImageJ to quantify monocyte–endothelial interactions. Adherent monocytes were enumerated at 1, 2, 3, 5, and 10 minutes post-perfusion using the Cell Counter plugin; adherent cells were defined as those remaining bound to the monolayer for >15 seconds (“Type 1” events). Each imaging field was divided into four equal quadrants, and cell counts from all regions were pooled for statistical evaluation. Data were expressed as mean ± SEM. For temporal resolution of diapedesis phases, start and endpoint times of individual transmigration events were recorded by manual tracking, and key intervals including adhesion-to-transmigration duration were calculated to assess treatment effects. Following transmigration, monocytes that entered the abluminal (sub-endothelial) compartment were identified by their transition from a phase-gray (luminal) to a phase-dark appearance under phase-contrast microscopy, as originally described by Bradfield et al. (11). Abluminal retention time was defined as the interval from the completion of TEM (first frame of fully phase-dark appearance) to the earliest of: (i) reverse transendothelial migration (rTEM; re-acquisition of a phase-bright/gray appearance indicating return to the luminal compartment), or (ii) the end of the 120-minute recording window. Cells that did not undergo rTEM within the recording window were classified as abluminally retained; cells that returned to the luminal surface were classified as abluminally egressed, and their abluminal dwell time was recorded accordingly. Cell death was distinguished from rTEM by the absence of directional movement followed by substrate detachment; such events were excluded from post-TEM analysis. All cell counts are expressed as the number of monocytes per imaging field of view (one field = one 20× objective field captured by the ZEISS AxioObserver; field area ~0.34 mm²), not as a fraction of the total monocyte suspension perfused through the channel. Four non-overlapping fields per channel were acquired per experiment, and values were averaged across fields prior to statistical analysis. This per-field quantification is the established standard for live-cell flow-based transmigration assays and is identical to the analytical unit reported by Bradfield et al. (11), in which comparable values of 20–60 monocytes per field were reported under equivalent conditions. Individual monocyte trajectories were tracked using the Manual Tracking plugin in ImageJ and exported for graphical representation and statistical comparison between experimental groups.

### 2.12 Statistical analysis

For experiments comparing diabetic and healthy monocytes, the Mann–Whitney Rank Sum Test or Kruskal–Wallis ANOVA on Ranks with Tukey or Dunn’s *post hoc* correction was used. Non-parametric rank-based tests were selected for these comparisons for the following reasons: (i) the sample sizes in these comparisons (n = 5–6 per group) are insufficient to reliably establish normality by standard tests such as Shapiro–Wilk; (ii) the distributions of several monocyte functional parameters showed visible skew or outliers at this sample size; and (iii) the use of non-parametric tests represents the conservative and appropriate default for small clinical sample comparisons in this context. For all other experiments, two-sample independent t-tests or Kruskal–Wallis ANOVA with *post hoc* correction was performed as appropriate. Statistical significance was defined as P < 0.05. Analyses were performed using SigmaPlot and GraphPad Prism 8 software.

### 2.13 Study participants and ethical approval

This study was approved by the ethics committee of Münster University Hospital (approvalnumber 2011-612-f-S) and conforms to the Declaration of Helsinki. All T2DM patients and non-diabetic control individuals providing blood for serum preparation provided written informed consent to participate. These donors were screened prior to enrolment using a structured health questionnaire reviewed by a trained study nurse. Non-diabetic control donors were excluded if they met any of the following criteria: (i) known diagnosis of diabetes mellitus (type 1 or type 2), impaired fasting glucose (≥5.6 mmol/L), or metabolic syndrome; (ii) active inflammatory, autoimmune, or infectious disease at the time of donation; (iii) current use of immunomodulatory, anti-inflammatory (including NSAIDs), lipid-lowering (statins), or antidiabetic medications; (iv) current or recent (within 3 months) smoking; (v) BMI >30 kg/m²; or (vi) personal history of cardiovascular disease, including myocardial infarction, stroke, heart failure, or peripheral arterial disease. Eligible non-diabetic donors were confirmed to have fasting plasma glucose <5.6 mmol/L and HbA1c <5.7% at the time of enrolment, verified from recent clinical records or measured on the donation day. Patient and donor characteristics are described in detail in **Supplementary Table 2**.

Peripheral blood for monocyte isolation was obtained separately from anonymous healthy donors via thrombocyte reduction system (TRS) filters provided by the blood bank of University Hospital Münster. These donors were screened according to standard blood bank institutional donation criteria; no personal identifying information was available to the research team. Human umbilical vein endothelial cells (HUVECs) were isolated from three healthy donors according to the Declaration of Helsinki and approved by the ethics boards of the University of Münster (2009-537-f-S).

## 3 Results

### 3.1 TNF-α and T2DM serum produce comparable monocyte adhesion but divergent transendothelial migration under physiological flow

CD14^+^ monocytes were perfused over HUVEC monolayers activated with TNF-α (10 ng/mL, 6 h) or T2DM patient serum (20% v/v, 6–24 h), and adhesion and TEM dynamics were recorded by real-time live-cell imaging over 30 minutes. This approach enables simultaneous quantification of rolling, firm adhesion, and transmigration under physiologically relevant hemodynamic conditions that recapitulate the postcapillary venule microenvironment (21, 42).

**Figure 1:**
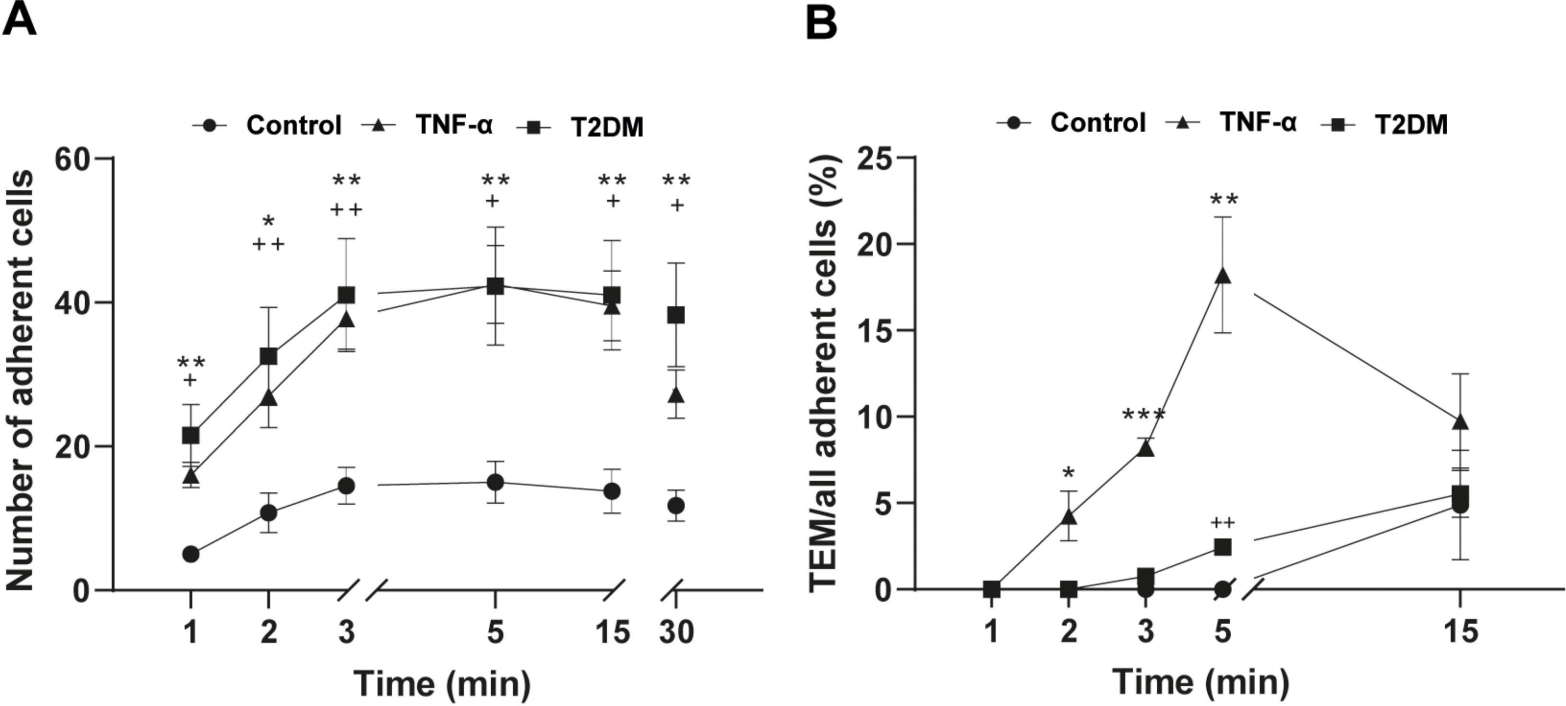
TNF-α and T2DM serum produce comparable monocyte adhesion but divergent transendothelial migration (TEM) under physiological flow. CD14^+^ monocytes were perfused over HUVEC monolayers activated with TNF-α (10 ng/mL, 6 h) or T2DM patient serum (20% v/v, 6–24 h), and adhesion/TEM dynamics were recorded by real-time live-cell imaging over 30 minutes under postcapillary venule shear conditions. The control condition consisted of HUVEC monolayers incubated in matched endothelial medium without cytokine or serum supplementation for the same duration. Data shown are from a representative experiment from one donor; similar results were obtained from 6 independent donors. **(A)** Time-course of firmly adherent monocytes per imaging field (four-quadrant count): control remains low (5–15 cells), whereas both TNF-α and T2DM reach ~40–42 adherent cells by 2–3 minutes and remain elevated. **(B)** TEM efficiency over time: control shows minimal TEM (<1%); TNF-α induces rapid TEM peaking at ~18% by 5 minutes; T2DM serum produces markedly impaired TEM reaching only ~5% by 15 minutes despite comparable adhesion. Statistical significance symbols: *p < 0.05 and p < 0.01 indicate significance relative to the unstimulated control condition; +p < 0.05 and ++ p < 0.01 indicate significance relative to the TNF-α condition. Values represent mean ± SEM.

Under control conditions, monocyte adhesion remained consistently low (5–15 cells) throughout the observation period (**Figure 1A**). TNF-α–activated endothelium demonstrated rapid and robust enhancement of monocyte adhesion, increasing from ~17 cells at 1 minute to ~30 cells by 2 minutes and reaching peak levels of ~40–42 adherent cells between 3 and 15 minutes (**Figure 1A**). T2DM serum-treated endothelium exhibited remarkably similar adhesion kinetics, beginning at ~22 cells at 1 minute and reaching ~40 cells by 2–3 minutes, maintained throughout the observation period (**Figure 1A**).

Despite comparable adhesion, TEM efficiency differed strikingly (**Figure 1B**). Control conditions showed minimal TEM (<1%). TNF-α–activated endothelium promoted rapid TEM, peaking at ~18% by 5 minutes before declining (**Figure 1B**). T2DM serum-treated endothelium showed markedly impaired TEM, reaching only ~5% by 15 minutes (**Figure 1B**). This dissociation—equivalent adhesion (~40 cells) but dramatically divergent TEM (18% vs. 5%)—constitutes the central functional finding and indicates that T2DM conditions specifically impair transmigration despite enhancing adhesive interactions. This observation is consistent with the multi-step adhesion cascade model, wherein firm adhesion and transmigration are independently regulated events requiring distinct molecular signals (21, 43).

### 3.2 TNF-α speeds up the process of adhesion-to-TEM transition and sustains transmigration over time

**Figure 2:**
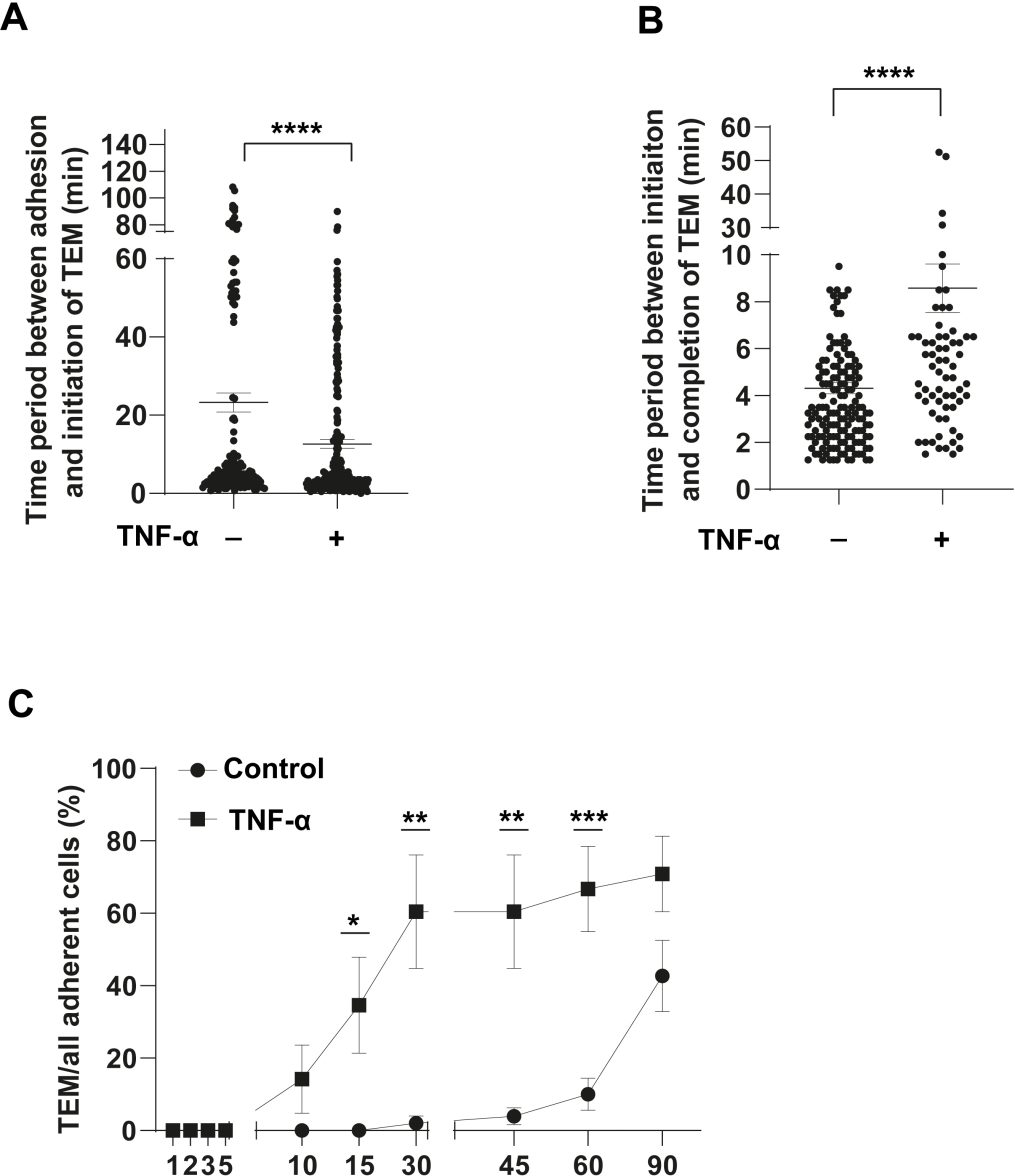
TNF-α accelerates the adhesion-to-TEM transition, increases TEM completion efficiency, and sustains transmigration over time. Single-cell kinetic parameters were extracted from tracked monocytes under TNF-α stimulation (10 ng/mL, 6 h) versus the unstimulated medium control (HUVEC in matched endothelial medium without cytokine). Data shown are from a representative experiment from one donor; similar results were obtained from 4 independent donors across 4 independent experiments. **(A)** Time from initial adhesion to TEM initiation: TNF-α significantly shortens the adhesion-to-TEM interval compared to unstimulated control (****p < 0.0001), with the majority of TNF-α-stimulated cells initiating TEM within 0–20 minutes, whereas control cells show prolonged delays of up to 120 minutes. **(B)** TEM completion time: TNF-α-stimulated monocytes complete transmigration significantly faster than control monocytes (****p < 0.0001), with most cells completing TEM within 2–8 minutes after initiation. **(C)** Fraction of wall-adherent cells undergoing TEM across 90 minutes: control remains near 0% throughout early time points, while TNF-α shows a progressive rise that is significantly greater than control from 15 minutes onward, reaching 60–70% by 30–45 minutes and approximately 70% by 90 minutes. Statistical significance between TNF-α and control conditions in panel C: *p < 0.05, **p < 0.01, ***p < 0.001. Values represent mean ± SEM.

The time between the first monocyte adhesion and the start of TEM showed that most of the control monocytes (about 95%) had long delays before starting TEM, with most of them needing 60–120 minutes (**Figure 2A**). On the other hand, TNF-α activation dramatically accelerated this transition: about 80% of adherent monocytes started to move within 0–20 minutes of initial adhesion (**Figure 2A**). This speed-up is in line with the quick NF-κB–mediated upregulation of ICAM-1, VCAM-1, and junctional adhesion molecules, which lower the threshold for productive monocyte–endothelial junctional engagement (44).

The activation of TNF-α made the completion of TEM much faster, with most monocytes completing transmigration within 2 to 8 minutes of starting (**Figure 2B**). This means that the junctions were remodeled in a way that allowed paracellular passage without any problems (42, 43). **Figure 2C** shows that control conditions kept TEM fractions low (<5%) for the whole 90-minute analysis. When TNF-α was activated, the cumulative TEM fraction showed a quick, steady rise that reached approximately 60–70% by 30–45 minutes and remained elevated for 90 minutes (**Figure 2C**).

### 3.3 T2DM serum recapitulates the shortened adhesion-to-TEM initiation time but not the overall TEM output of TNF-α

**Figure 3:**
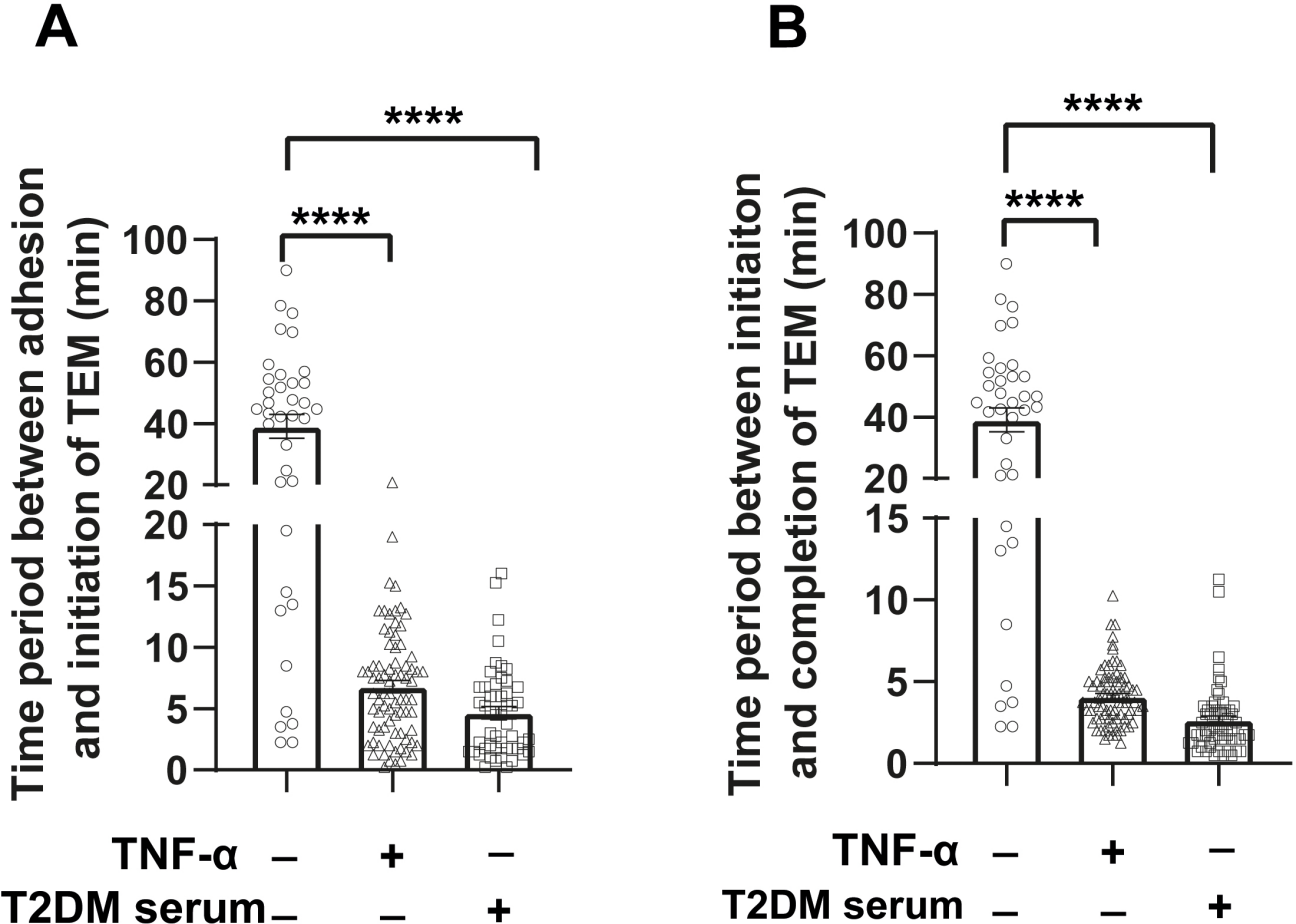
T2DM serum reproduces shortened single-cell TEM initiation and TEM phase duration but not the population-level TEM output induced by TNF-α. Monocytes were tracked under control (unstimulated HUVEC in matched endothelial medium), TNF-α (10 ng/mL, 6 h), and T2DM serum (20% v/v, 6–24 h) stimulation. Data shown are from a representative experiment from one donor; similar results were obtained from 4 independent donors across 4 independent experiments. **(A)** Adhesion-to-TEM initiation interval: both TNF-α and T2DM serum significantly reduce the time between initial monocyte adhesion and TEM initiation compared with the unstimulated control (****p < 0.0001 for each vs. control); the two stimulated conditions also differ significantly from each other (****p < 0.0001). **(B)** TEM phase duration: both TNF-α and T2DM serum similarly and significantly shorten the duration of the active transmigration phase relative to control (****p < 0.0001 for each vs. control), indicating comparable per-cell crossing kinetics once TEM is engaged; no significant difference was detected between TNF-α and T2DM serum conditions. Note that these single-cell kinetic parameters reflect the speed of individual transmigration events among monocytes that successfully engaged the endothelial junction, and should not be interpreted as reflecting overall population-level TEM efficiency, which is quantified separately in **Figure 2C** and differs substantially between TNF-α and T2DM serum conditions. Values represent mean ± SEM.

It is important to distinguish between two conceptually separate parameters measured in this study: the per-cell kinetic speed of individual transmigration events (i.e., how long it takes a single monocyte to complete TEM once it has engaged the endothelial junction; **Figures 2**, **3**), and the population-level TEM efficiency (i.e., the proportion of all adherent monocytes that successfully initiate and complete TEM, **Figure 2C**). Both TNF-α and T2DM serum reduce the per-cell adhesion-to-TEM interval relative to unstimulated control (**Figures 3A, B**), indicating that once a monocyte engages the transmigration machinery, early TEM entry cues are active under both stimuli. However, population-level TEM efficiency differs markedly between the two conditions (~18% under TNF-α vs. ~5% under T2DM serum; **Figure 2C**), reflecting a profound difference in the proportion of adherent monocytes that successfully reach the transmigration step, rather than in the speed of individual crossing events.

We next directly compared TNF-α versus T2DM serum versus control for both the adhesion-to-TEM initiation interval and the TEM phase duration. Both TNF-α and T2DM serum significantly reduced the time from adhesion to TEM initiation compared with control (**Figure 3A**). This indicates that the diabetic milieu is sufficient to trigger early TEM entry cues under flow, similar to classical inflammatory activation.

Similarly, both TNF-α and T2DM serum significantly shortened the TEM phase duration relative to control (**Figure 3B**). The duration of active transmigration was comparable between the two stimulation conditions, suggesting that junctional remodeling and diapedesis mechanics are not selectively impaired in the diabetic milieu.

However, this similarity in single-cell initiation and completion kinetics contrasts sharply with the markedly reduced net TEM output under T2DM conditions observed in **Figure 1B** (5% vs 18% for TNF-α). This dissociation between similar per-cell timing and dramatically different population-level TEM points to differences in the number of monocytes that successfully engage the transmigration machinery, rather than in the kinetics of individual crossing events.

### 3.4 Hyperglycemia- and T2DM-treated endothelium promotes prolonged abluminal monocyte retention, while TNF-α accelerates egress

**Figure 4:**
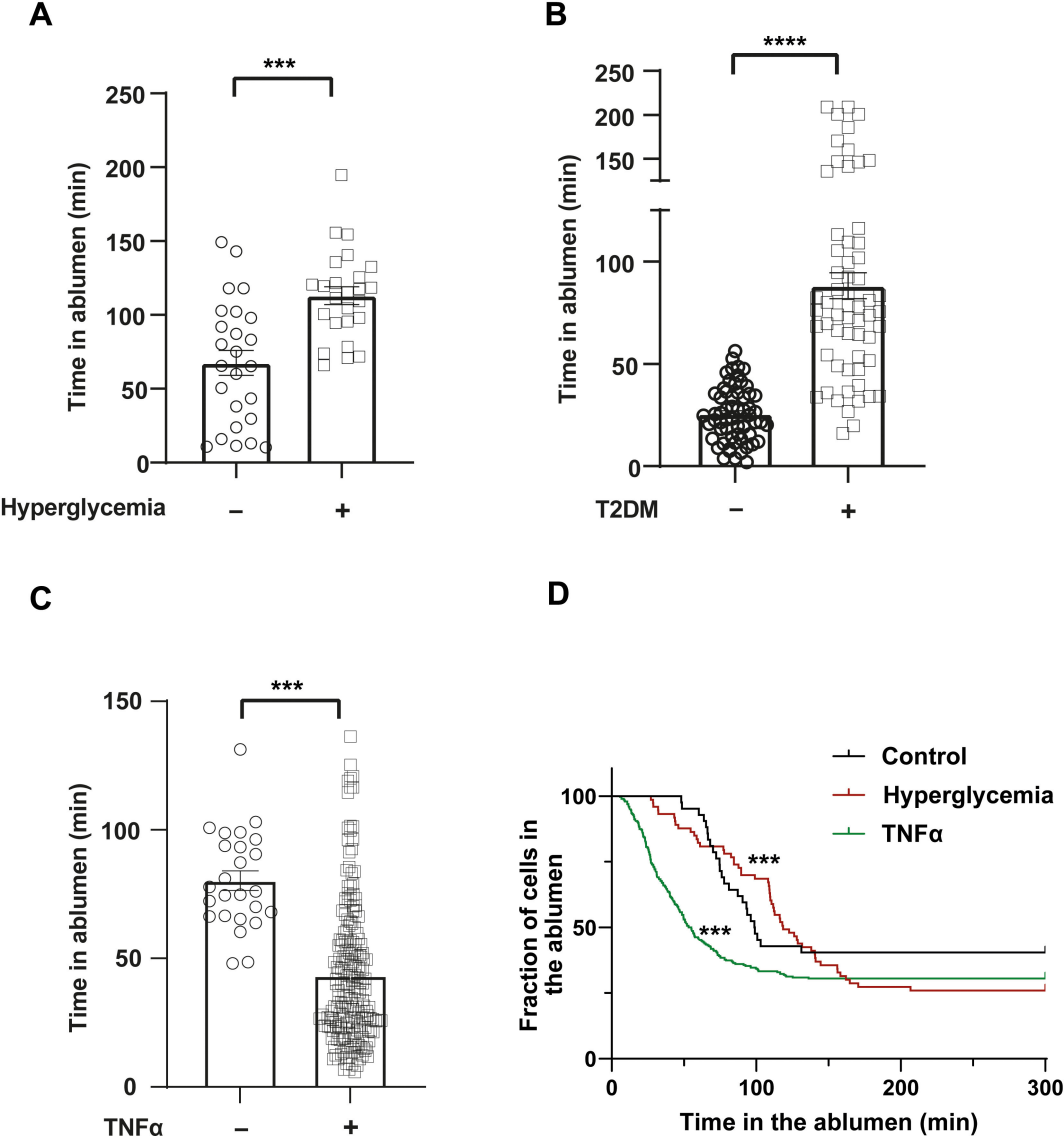
Hyperglycemia and T2DM imprint prolonged abluminal retention on monocytes, whereas TNF-α accelerates abluminal egress. Following transendothelial migration (TEM), monocytes residing in the abluminal compartment were identified by their phase-dark appearance under phase-contrast microscopy. Reverse TEM (rTEM), the return of abluminal monocytes to the luminal surface, was identified by re-acquisition of a phase-bright/gray appearance and confirmed by directional movement back across the endothelial junction. Abluminal retention time was defined as the interval from TEM completion to rTEM initiation, or to the end of the 120-minute recording window for cells that did not undergo rTEM. Data shown in panels A–C are from a representative experiment from one donor; similar results were obtained from 4 independent donors across 4 independent experiments. Panel D is derived from n = 3 independent experiments. **(A)** Endothelial cells exposed to hyperglycemia (25 mM glucose, 48 h) display significantly increased abluminal residence time compared with normoglycemic controls (5 mM glucose, matched duration; ***p < 0.001). **(B)** Endothelial cells treated with T2DM patient serum (20% v/v, 6–24 h) show markedly and significantly prolonged abluminal residence compared with HUVECs treated with age-matched non-diabetic donor serum at the same concentration and duration (****p < 0.0001). **(C)** TNF-α activation (10 ng/mL, 6 h) of the endothelial monolayer significantly reduces abluminal residence time relative to unstimulated HUVEC in matched medium without cytokine, consistent with enhanced rTEM (***p < 0.001). **(D)** Kaplan–Meier-style survival analysis of abluminal residence time across all three conditions (n = 3 independent experiments): TNF-α-stimulated endothelium supports the most rapid abluminal egress; unstimulated medium control displays intermediate kinetics; hyperglycemia-treated endothelium shows significantly prolonged abluminal retention compared with both control and TNF-α conditions (***p < 0.001 for each comparison). Prolonged abluminal retention under diabetic and hyperglycemic conditions reflects impaired rTEM rather than cell death, as non-viable cells detach from the substrate and are excluded from analysis. Values represent mean ± SEM.

Because impaired TEM alone did not fully explain the trafficking differences, we quantified the time spent by monocytes in the abluminal compartment following transmigration as a readout of post-TEM fate. Endothelial cells exposed ex vivo to hyperglycemic conditions (25 mM glucose for 48 hours) demonstrated a significant increase in abluminal accumulation time for monocytes compared with normoglycemic controls (5 mM glucose) (p<0.001; **Figure 4A**). This finding indicates that hyperglycemia can directly reprogram monocyte post-transmigration behavior. When endothelial cells were exposed to serum from T2DM patients, they showed an even more pronounced prolongation of abluminal residence time compared with serum from healthy donors (p<0.0001; **Figure 4B**). The marked increase in monocyte abluminal time demonstrates that the diabetic serum milieu induces endothelial changes that promote prolonged monocyte retention. This prolonged abluminal retention is consistent with impaired reverse transendothelial migration (rTEM) and establishes a retention-based rather than recruitment-based mechanism of monocyte accumulation in diabetic conditions (10).

In striking contrast, TNF-α activation of the endothelial monolayer significantly reduced the time monocytes spent in the abluminal compartment compared with unstimulated controls (p<0.001; **Figure 4C**). This accelerated abluminal egress under TNF-α conditions indicates that acute inflammatory endothelial activation not only promotes efficient transmigration (**Figure 2**) but also facilitates the subsequent reverse transendothelial migration of monocytes, consistent with an organized junctional remodeling program that supports bidirectional monocyte passage.

To directly compare the abluminal residence dynamics across all three conditions, we performed Kaplan–Meier-style survival analysis plotting the fraction of cells remaining in the ablumen over time (**Figure 4D**). TNF-α-stimulated endothelium supported the most rapid monocyte egress, with the majority of transmigrated cells exiting the abluminal compartment within the first 50–75 minutes. Monocytes on control (normoglycemic) endothelium displayed intermediate kinetics, whereas monocytes on hyperglycemia-treated endothelium exhibited a markedly right-shifted survival curve, indicating significantly prolonged abluminal retention (p<0.001 vs. both control and TNF-α). Both the TNF-α and hyperglycemia curves eventually plateau at approximately 20–30% of cells remaining in the ablumen by 200–300 minutes, suggesting a subpopulation of cells that fails to reverse transmigrate regardless of conditions; however, the kinetics of egress for the remaining population differ dramatically between conditions.

Collectively, these data establish that TNF-α and T2DM/hyperglycemia exert opposing effects on monocyte post-transmigration fate: TNF-α accelerates abluminal egress consistent with an acute inflammatory response that resolves through efficient monocyte trafficking, whereas hyperglycemic and diabetic conditions prolong abluminal retention through impaired rTEM, promoting chronic monocyte accumulation characteristic of diabetic atherogenesis (10, 45).

### 3.5 Transcriptomic analysis reveals distinct molecular programs underlying T2DM versus TNF-α endothelial activation

To clarify the molecular foundation for the observed dissociation between adhesion and transendothelial migration (TEM), publicly accessible bulk RNA-seq datasets were examined, comparing endothelial cells from T2DM patients (T2DM dermal ECs; GSE92724, n=4 T2DM vs. n=6 healthy) and TNF-α–stimulated endothelial cells (TNF-α coronary artery ECs; GSE134489, n=3 TNF-α vs. n=3 basal).

**Figure 5:**
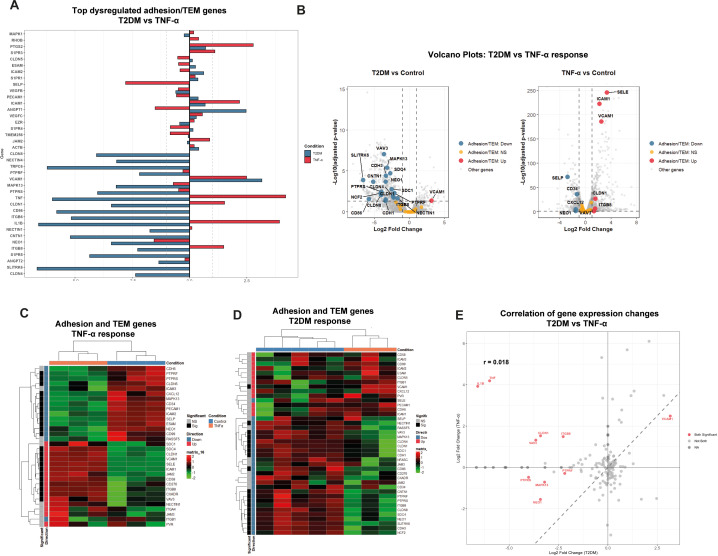
Transcriptomic analysis reveals distinct molecular programs underlying T2DM versus TNF-α endothelial activation. Public bulk RNA-seq datasets were analyzed comparing T2DM endothelial cells (GSE92724; n=4 T2DM vs. n=6 healthy) and TNF-α–stimulated endothelial cells (GSE134489; n=3 TNF-α vs. n=3 basal), focusing on adhesion, junctional, and TEM-related genes. **(A)** Side-by-side horizontal bar chart of top dysregulated adhesion/TEM genes highlights divergent expression profiles between conditions. Log_2_ fold-changes calculated independently within each dataset relative to its own matched control (T2DM: diabetic vs. normoglycemic ECs, GSE92724; TNF-α: stimulated vs. vehicle-treated HCAECs, GSE134489). Values reflect stimulus-specific transcriptional responses and do not represent cross-dataset absolute expression comparisons. **(B)** Volcano plots show mixed up-/downregulation with moderate fold changes in T2DM, versus robust, highly significant induction of multiple adhesion/TEM genes under TNF-α. **(C)** Heatmap of TNF-α samples shows consistent upregulation of VCAM1, ICAM1, SELE, JAM2, and JAM3. **(D)** Heatmap of T2DM samples shows heterogeneous regulation with VCAM1 upregulation, downregulation of tight junction genes (CLDN1, CLDN4, CLDN5), and minimal ICAM1 change. **(E)** Pearson correlation of log_2_ fold-changes between the TNF-α dataset (GSE134489; TNF-α vs. vehicle control) and the T2DM dataset (GSE92724; diabetic vs. normoglycemic control). Each dataset was independently normalized to its own matched control prior to comparison; the correlation therefore reflects similarity in transcriptional response rather than absolute expression levels. r = 0.018 indicates near-zero transcriptional overlap between the two stimuli. Each point represents one gene detected as differentially expressed in at least one dataset.

The side-by-side horizontal bar chart of the top dysregulated adhesion/TEM genes (**Figure 5A**) revealed markedly divergent expression profiles between the two conditions. Volcano plot analysis (**Figure 5B**) further highlighted these differences: the T2DM versus control comparison showed moderate bidirectional fold changes across adhesion and junction-related transcripts, whereas the TNF-α versus control comparison was characterized by strong, statistically significant upregulation of a discrete set of adhesion and TEM genes.

The heatmap analysis (**Figures 5C, D**) consistently showed that VCAM1, ICAM1, SELE, JAM2, and JAM3 were all upregulated in TNF-α samples. This is because NF-κB-driven programs coordinate adhesion molecule upregulation with junctional remodeling at the same time (44, 46). The T2DM heatmap (**Figure 5D**) showed a mixed pattern: VCAM1 was upregulated while several tight junction genes (CLDN1, CLDN4, CLDN5) were downregulated, and there was only a small change in ICAM1.

To assess transcriptional overlap between the two stimuli, differentially expressed genes from each dataset were first normalized to their own internally matched control condition — normoglycemic donor cells for GSE92724 and vehicle-treated HCAECs for GSE134489 — and log_2_ fold-changes were calculated independently within each dataset prior to any cross-dataset comparison. Pearson correlation of these log_2_ fold-changes (**Figure 5E**) revealed a near-zero relationship (r = 0.018), indicating that TNF-α and T2DM drive largely non-overlapping transcriptional programs despite both promoting monocyte–endothelial adhesion at the functional level, and providing direct evidence that the two stimuli activate endothelium through fundamentally distinct molecular pathways.

**Figure 6:**
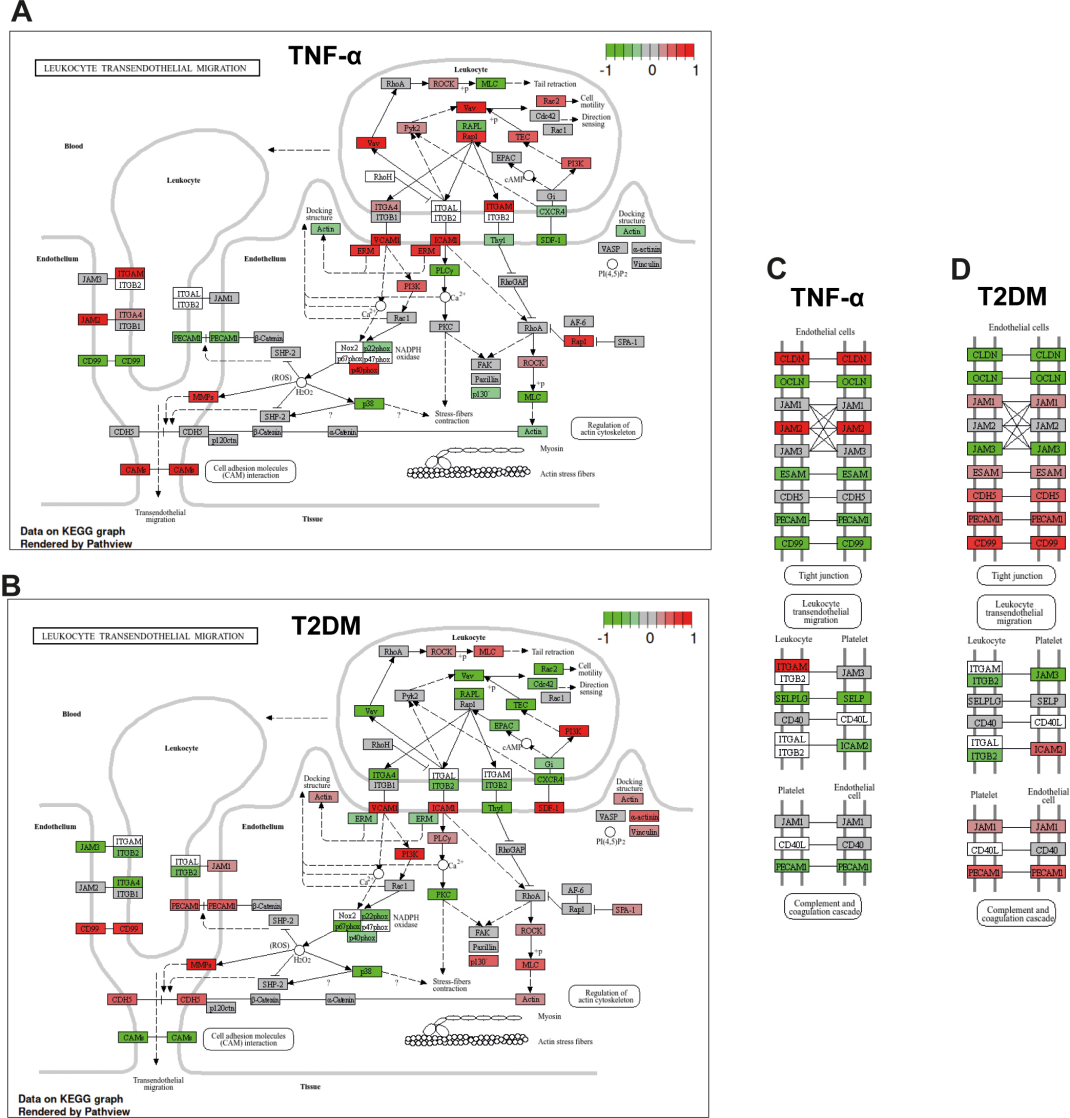
KEGG pathway overlays reveal coordinated TNF-α activation versus T2DM-associated dysregulation in leukocyte transmigration and cell-adhesion programs. Pathview overlays were generated from RNA-seq differential expression for TNF-α and T2DM conditions. Red indicates upregulation and green indicates downregulation. **(A)** Leukocyte transendothelial migration (TEM) KEGG pathway overlay under TNF-α stimulation shows coordinated activation across junctional and migration modules (including tight-junction/junctional components and leukocyte-side migratory machinery) consistent with permissive paracellular TEM. **(B)** Leukocyte TEM KEGG pathway overlay under T2DM shows an opposing/dysregulated pattern characterized by reduced tight-junction module expression and altered junctional architecture consistent with impaired TEM. **(C)** Cell adhesion molecule (CAM) interaction KEGG pathway overlay under TNF-α demonstrates broad induction of adhesion modules that support leukocyte recruitment and productive endothelial–leukocyte interactions. **(D)** CAM interaction KEGG pathway overlay under T2DM shows a distinct, non-uniform CAM signature (selective induction of subsets of adhesion nodes without a fully coordinated recruitment program), consistent with strong adhesion but inefficient downstream transmigration.

GO enrichment analysis (**Supplementary Figure 3**) confirmed and extended these findings at the pathway-ontology level. In TNF-α–stimulated endothelial cells (GSE134489), GO-BP terms were dominated by leukocyte cell-cell adhesion, leukocyte proliferation, myeloid leukocyte differentiation, and extracellular matrix organization (**Supplementary Figure 3A**), consistent with coordinate pro-inflammatory endothelial activation (consistent with the NF-κB pathway enrichment shown in **Figure 6**). GO-MF highlighted cytokine receptor binding, cytokine activity, and TNF receptor superfamily binding (**Supplementary Figure 3C**), while GO-CC showed enrichment for the external side of the plasma membrane, membrane rafts, and cell-substrate junction compartments (**Supplementary Figure 3B**) — the subcellular compartments in which VCAM1, ICAM1, and E-selectin are surface-displayed to facilitate monocyte capture. In contrast, T2DM endothelial cells (GSE92724) showed GO-BP enrichment for cell-cell junction organization, cell-cell junction assembly, and cell-substrate adhesion (**Supplementary Figure 3A**) — consistent with junctional disorganization and structural endothelial remodeling rather than acute inflammatory activation — together with GO-MF enrichment for glycosaminoglycan binding, integrin binding, and cell adhesion mediator activity (**Supplementary Figure 3C**), and GO-CC enrichment for basolateral and basal plasma membrane compartments and the collagen-containing extracellular matrix (**Supplementary Figure 3B**), reflecting chronic basement membrane thickening and altered integrin-ECM crosstalk under metabolic stress. To formally quantify the divergence between the two programs at the ontology level, we performed a cross-condition GO term correlation analysis restricted to vascular- and immune-relevant terms across all three ontology domains (**Supplementary Figure 4B**). The Pearson correlation of −log10(adjusted p-value) enrichment scores between T2DM and TNF-α was r = 0.157 (n = 66 shared terms), directly mirroring the near-zero transcriptome-level correlation (r = 0.018; **Figure 5E**) and confirming that these stimuli engage qualitatively non-overlapping molecular programs at the pathway-ontology level. GO Molecular Function enrichment further reinforced this conclusion: whereas TNF-α endothelial cells were characterized by cytokine receptor binding and TNF receptor superfamily activity, T2DM endothelial cells displayed gap junction channel activity, cell-cell adhesion mediator activity, and glycosaminoglycan binding (**Supplementary Figure 4A**), reflecting fundamentally different receptor-ligand interaction landscapes underlying each condition.

### 3.6 KEGG pathway overlays reveal organized TNF-α junctional remodeling versus T2DM junctional disorganization

To map transcriptomic changes onto functional signaling architecture, Pathview overlays of the Leukocyte Transendothelial Migration KEGG pathway were generated for both conditions (**Figure 6**). In the TNF-α condition (**Figure 6A**), multiple nodes across both junctional and migratory modules were upregulated, including claudins, occludin (OCLN), JAM1–JAM3 in the endothelial tight junction compartment, and ITGAM (CD11b) and ITGB2 (CD18) on the leukocyte side. CDH5 (VE-cadherin) was downregulated, consistent with controlled junctional opening that facilitates paracellular TEM (43, 47). In the T2DM condition (**Figure 6B**), claudins, JAM1, JAM2, and JAM3 were downregulated, whereas CDH5 was paradoxically upregulated. Leukocyte ITGAM and ITGB2 were also suppressed. This disorganized pattern—characterized by downregulated tight junction components coupled with stabilized adherens junctions—creates a rigid endothelial barrier that severely impedes paracellular monocyte passage despite robust VCAM1-mediated adhesion. The concurrent loss of tight junction proteins and stabilization of adherens junctions represents the opposite of what is required for effective paracellular TEM, wherein VE-cadherin must be transiently displaced to permit leukocyte passage (47, 48).

**Figure 7:**
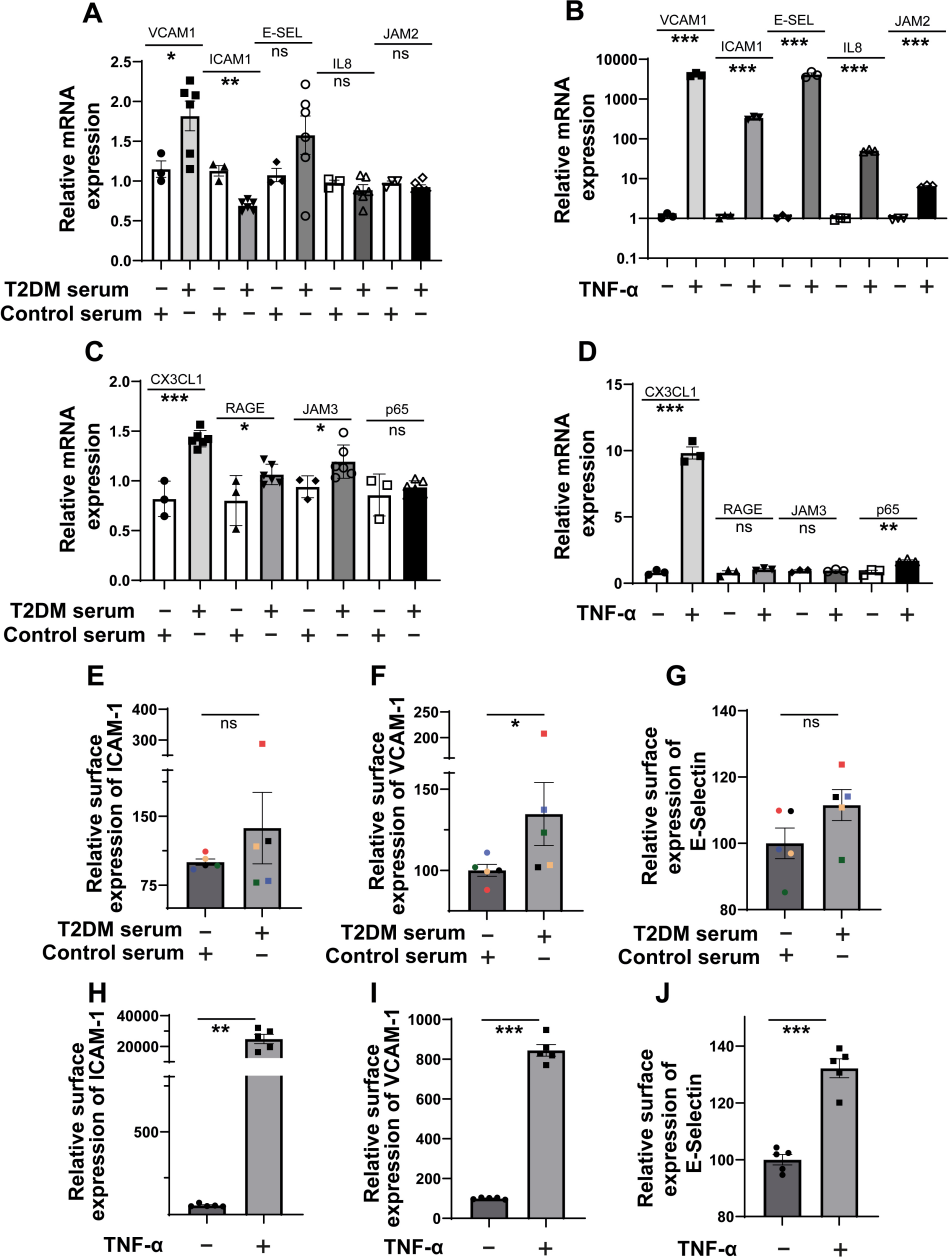
qPCR and flow cytometry validation confirm VCAM1-biased, ICAM1-deficient endothelial activation in T2DM versus coordinated inflammatory surface induction by TNF-α. HUVECs were treated with T2DM serum (20% v/v, 6 h) or TNF-α (10 ng/mL, 6 h) and assessed by qPCR **(A–D)** or flow cytometry for surface protein expression **(E–J)**. For qPCR, all conditions were normalized to the respective unstimulated control (set to 1.0); note logarithmic scale in **(B)** and **(D)**. **(A)** T2DM serum induces significant VCAM1 upregulation and significant ICAM1 downregulation, while E-selectin, IL-8, and JAM2 show no significant change. Each data point represents one independent biological replicate (n = 6 T2DM serum donors; n = 3 non-diabetic control donors). **(B)** TNF-α induces coordinated and markedly greater upregulation of VCAM1, ICAM1, E-selectin, IL-8, and JAM2. Each data point represents one independent experiment (n = 6). **(C)** Pathway-selective markers under T2DM serum: CX3CL1 and RAGE (AGER) are significantly induced; JAM3 is significantly upregulated; RELA/p65 shows no significant change. n = 6 T2DM serum donors; n = 3 non-diabetic control donors. **(D)** Pathway-selective markers under TNF-α: CX3CL1 and RELA/p65 are significantly induced; RAGE and JAM3 show no significant change. n = 6 independent experiments. **(E–G)** Surface protein expression under T2DM serum stimulation quantified by flow cytometry: ICAM-1 surface levels are not significantly changed **(E)**; VCAM-1 surface levels are significantly increased **(F)**; E-Selectin surface levels are not significantly changed **(G)**. Each symbol color represents an individual serum donor (n = 5 T2DM donors; n = 5 non-diabetic control donors), allowing cross-panel tracking of individual donor responses. **(H–J)** Surface protein expression under TNF-α stimulation: ICAM-1 **(H)**, VCAM-1 **(I)**, and E-Selectin **(J)** are all significantly and markedly induced at the cell surface. n = 5 independent experiments. Values represent mean ± SEM. Statistical significance: *p < 0.05, **p < 0.01, ***p < 0.001; ns, not significant.

To complement the Leukocyte TEM pathway analysis, Pathview overlays of the Cell Adhesion Molecule (CAM) Interaction KEGG pathway were generated for both conditions (**Figures 6C, D**). Under TNF-α stimulation (**Figure 6C**), coordinate upregulation of ICAM1, VCAM1, SELE, and PECAM1 on endothelial cells was observed alongside upregulation of their leukocyte counter-receptors (ITGAL/LFA-1, ITGAM/MAC-1), reflecting balanced multi-receptor engagement that supports the complete adhesion cascade from selectin-mediated rolling through integrin-dependent arrest and junctional passage. In contrast, the T2DM CAM pathway overlay (**Figure 6D**) revealed selective upregulation of VCAM1 and PECAM1, alongside induction of ALCAM and CD40, but without coordinate leukocyte integrin upregulation. Notably, although the KEGG Pathview overlay depicted ICAM1 and SELE as upregulated at the transcriptomic level based on the T2DM RNA-seq dataset (GSE92724, comprising dermal endothelial cells from T2DM patients), qPCR validation in T2DM serum-treated HUVECs confirmed significant ICAM1 downregulation (0.61-fold, p<0.001; **Figure 7A**), suggesting context-dependent regulation that may reflect differences between chronic *in vivo* diabetic endothelium and acute ex vivo serum treatment, or tissue-specific variation between dermal and umbilical vein endothelial cells. Irrespective of this discrepancy, the functional outcome converges: T2DM endothelial activation produces an adhesion molecule profile that promotes monocyte tethering and firm adhesion through VCAM1–VLA-4 interactions, while lacking the integrin co-activation on the leukocyte side required for efficient junctional passage—consistent with the adhesion–TEM dissociation observed in **Figure 1**.

### 3.7 qPCR and flow cytometry validation confirm VCAM1-biased, ICAM1-deficient endothelial activation in T2DM versus coordinate inflammatory activation by TNF-α

To confirm the RNA-seq results in the experimental HUVEC system, quantitative PCR and flow cytometric surface protein quantification were conducted on control, TNF-α–stimulated (10 ng/mL, 6 h), and T2DM patient serum-treated (20% v/v, 6 h) HUVECs, assessing the expression of critical adhesion molecules, junctional proteins, inflammatory mediators, and metabolic stress markers. It should be noted that all qPCR analyses were performed under static culture conditions. While shear stress is known to independently modulate endothelial gene expression — including downregulation of pro-inflammatory adhesion molecules and upregulation of atheroprotective transcription factors such as KLF2 and eNOS — the static conditions used here provide mechanistic insight into stimulus-specific transcriptional responses in isolation and are interpreted as complementary to rather than reflective of the full flow-condition transcriptome. The potential confounding effect of shear stress on endothelial gene expression under flow is acknowledged as a limitation of this analysis (see Section 4, Limitations).

TNF-α stimulation induced a robust, coordinate upregulation of all three main adhesion molecules — VCAM1, ICAM1, and E-selectin — as well as a strong increase in IL-8 and JAM2 at the mRNA level (**Figure 7B**). Among pathway-selective markers, CX3CL1 (fractalkine) and RELA/p65 exhibited highly significant upregulation, whereas RAGE and JAM3 showed no significant change (**Figure 7D**). The coordinate transcriptional induction was fully reflected at the protein level: flow cytometric quantification confirmed significant upregulation of ICAM-1, VCAM-1, and E-Selectin surface expression following TNF-α stimulation (**Figures 7H–J**), consistent with the broad pro-inflammatory surface remodeling that supports leukocyte capture and transmigration.

T2DM serum produced a qualitatively distinct profile. At the mRNA level, VCAM1 was upregulated while ICAM1 was significantly downregulated (0.61-fold, p < 0.001), and E-selectin, IL-8, and JAM2 showed no significant change (**Figure 7A**). Among pathway-selective markers, CX3CL1 and RAGE were significantly induced, JAM3 was significantly upregulated, and RELA/p65 remained unchanged (**Figure 7C**). Critically, this mRNA dissociation between VCAM1 and ICAM1 was partially mirrored at the protein level: VCAM-1 surface expression was significantly increased (**Figure 7F**), whereas ICAM-1 and E-Selectin surface levels did not reach statistical significance (**Figures 7E, G**), corroborating the selective VCAM1-dominant surface activation pattern under T2DM conditions. The specific induction of RAGE substantiates AGE-RAGE signaling within the diabetic context (27, 28). The ICAM1 deficiency is mechanistically critical because ICAM1–LFA-1 interactions are essential for junctional passage during TEM, and ICAM1 cytoplasmic domain signaling through Rho GTPases is required to initiate junctional opening (43, 49). The upregulation of JAM3 in T2DM aligns with compromised rTEM via JAM-3–MAC-1 interactions, leading to extended abluminal retention (10, 11). The lack of p65 induction distinguishes the T2DM metabolic-inflammatory program from classical NF-κB-driven acute inflammatory activation (44, 46).

## 4 Discussion

**Figure 8:**
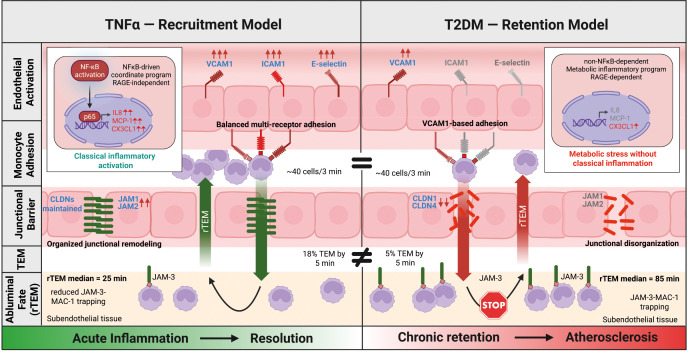
Integrated model of TNF-α–driven monocyte recruitment versus T2DM-driven monocyte retention. Schematic summarizing the two contrasting paradigms of monocyte–endothelial interaction. Left — TNF-α Recruitment Model: NF-κB–driven coordinate activation (p65↑, IL-8↑, CX3CL1↑) produces balanced adhesion molecule expression (VCAM1↑, ICAM1↑, E-selectin↑) engaging VLA-4, LFA-1, and PSGL-1 on monocytes. Organized junctional remodeling (JAM1↑, JAM2↑, claudins maintained) enables efficient TEM (~18% by 5 min) and rapid abluminal egress (median ~25 min), supporting inflammatory resolution. Right — T2DM Retention Model: T2DM-based signaling produces VCAM1-biased adhesion with junctional disorganization. Impaired TEM (~5% by 5 min) is compounded by JAM-3–MAC-1 trapping of transmigrated monocytes, producing prolonged abluminal retention (median ~85 min) and chronic subendothelial accumulation driving accelerated atherosclerosis.

The current research contributes additional, converging data that TNF-α and T2DM serum induce endothelial activation through distinct, mechanistic pathways, with fundamentally different outcomes for monocyte adhesion and transmigration. By employing a physiologically relevant flow imaging platform, transcriptomic analysis, GO enrichment analysis, and qPCR and flow cytometric verification, we have demonstrated that TNF-α triggers a highly coordinated NF-κB-dependent inflammatory response, which couples balanced adhesion molecule upregulation with organized junctional remodeling and facilitates highly efficient abluminal egress (**Figures 1**–**4**, **7B, D, H–J**). In contrast, T2DM serum triggers a disorganized endothelial phenotype, which exhibits highly efficient VCAM1-dependent adhesion, but ineffective transmigration, and is driven by AGE-RAGE signaling and junctional disorganization, culminating in monocyte retention and vascular inflammation (**Figures 1**–**7A, C**). The current model integrates these findings (**Figure 8**).

TNF-α stimulation elicited a significant, coordinated induction of VCAM1, ICAM1, and E-selectin, consistent with NF-κB and AP-1-dependent endothelial activation (44, 46). The coordinated induction of adhesion molecules is mechanistically important, since highly efficient monocyte transmigration under flow requires the sequential interaction of multiple adhesion molecules, including selectins, chemokines, integrins, and ICAM1/LFA-1 and VCAM1/VLA-4 (21, 43). The coordinated induction distinguishes acute inflammatory activation from the partial activation patterns that characterize endothelial activation under conditions of metabolic stress, in which adhesion molecules exhibit differential regulation (50, 51). TNF-α stimulation elicited rapid adhesion (40 cells/3 minutes), highly efficient transmigration (18% cells/5 minutes), and rapid abluminal egress (25 minutes, median time). The simultaneous induction of JAM2 (and JAM1 at the transcriptomic level) and claudin expression, along with selective VE-cadherin opening (**Figure 6A**), suggests organized junctional remodeling, rather than dysfunction, which is conducive to transendothelial migration while maintaining endothelial integrity (47, 49). The coordinated transcriptional response to TNF-α was fully recapitulated at the protein level: flow cytometric quantification of surface-expressed ICAM-1, VCAM-1, and E-Selectin confirmed broad and significant surface induction on TNF-α–stimulated HUVECs (**Figures 7H–J**), corroborating the mRNA data and affirming that the TNF-α–driven program results in comprehensive surface remodeling competent for all stages of the leukocyte adhesion cascade. GO enrichment analysis further substantiated this conclusion: at the pathway-ontology level, TNF-α endothelial cells were characterized by GO-BP terms related to leukocyte proliferation, leukocyte cell-cell adhesion, and myeloid leukocyte differentiation, GO-CC enrichment for the external side of the plasma membrane and membrane rafts, and GO-MF enrichment for cytokine receptor binding and TNF receptor superfamily activity — all consistent with a coordinate surface-directed pro-inflammatory program (**Supplementary Figures 3**, **S4**).

In stark contrast, T2DM serum induced a VCAM1-biased, ICAM1-deficient activation profile, as evidenced by RNA-seq data (GSE92724; **Figure 5D**). ICAM1 deficiency is mechanistically significant because ICAM1-LFA1 interactions are critical not only for strong adhesion but also for initiating junction opening during TEM; ICAM-1 cytoplasmic signaling via Src and Pyk2 kinases activates VE-cadherin Y658 and Y731 phosphorylation, events required for efficient TEM (43, 52). ICAM-1 expression at tricellular endothelial junctions *in vivo* identifies preferred routes of TEM, and ICAM-1 inhibition reduces TEM by approximately 69% (53). ICAM-1 engagement also activates endothelial RhoA-mediated cytoskeletal changes required for junctional opening (42, 54). Notably, while VCAM-1 dominates early atherogenesis—as demonstrated in LDLR-/- mice harboring a hypomorphic VCAM-1 allele, where VCAM-1 reduction but not ICAM-1 deficiency attenuated lesion formation (55)—this finding underscores that VCAM-1-mediated adhesion alone is insufficient to drive productive monocyte trafficking without ICAM-1 co-engagement. The selective VCAM1-dominant transcriptional profile was further corroborated at the surface protein level: flow cytometry demonstrated significant VCAM-1 surface upregulation under T2DM serum stimulation, whereas ICAM-1 and E-Selectin surface levels did not reach statistical significance (**Figures 7E–G**), directly mirroring the mRNA dissociation and confirming that the T2DM endothelial surface preferentially presents VCAM-1 while failing to mount a coordinate ICAM-1 and E-Selectin induction. At the pathway-ontology level, GO enrichment analysis of the T2DM transcriptome revealed a fundamentally distinct signature: GO-BP terms were dominated by cell-cell junction organization, cell-cell junction assembly, and cell-substrate adhesion, GO-CC enrichment highlighted basolateral and basal plasma membrane compartments and the collagen-containing extracellular matrix, and GO-MF enrichment identified glycosaminoglycan binding, integrin binding, and cell adhesion mediator activity (**Supplementary Figure 3**). These ontology-level findings are consistent with chronic junctional structural remodeling and altered ECM-integrin crosstalk rather than surface inflammatory activation and are entirely distinct from the TNF-α GO profile. Cross-condition GO term correlation analysis (r = 0.157, n = 66 shared terms; **Supplementary Figure 4**) directly mirrored the near-zero gene-level Pearson correlation (r = 0.018, **Figure 5E**), providing orthogonal pathway-level evidence that T2DM and TNF-α engage non-overlapping endothelial molecular programs.

KEGG overlay analysis (**Figure 6B**) revealed the downregulation of claudins and JAM1/JAM2, as well as the stabilization of VE-cadherin. Under physiological inflammatory conditions, the transient phosphorylation of VE-cadherin followed by its removal from the junction facilitates the transmigration of leukocytes (47, 52). Claudins and JAM family members, on the other hand, are junctional molecules that control junctional opening (43). These changes in junctional structure seen in T2DM correlate with the idea that the endothelial dysfunction caused by hyperglycemia regulates the endothelial barrier function through mechanisms fundamentally different from those engaged by classical inflammatory cytokines (48).

A striking observation is the identification of the distinct upstream signaling pathway of T2DM: AGE-RAGE signaling. RAGE was found to be specifically upregulated in HUVECs treated with T2DM serum (**Figure 7C**) but not TNF-α (**Figure 7D**), in contrast to p65. RAGE signaling activates PKC, p38 MAPK, and low-level NF-κB signaling, leading to distinct transcriptional outputs compared to TNF-α stimulation (28, 56). RAGE deficiency in diabetic ApoE−/− mice reduced the plaque area from 14.9% to 4.9% and attenuated leukocyte recruitment and VCAM-1 expression (57), thereby directly implicating RAGE in diabetic vascular inflammation. Furthermore, hyperglycemia is known to increase myelopoiesis via S100A8/A9 and RAGE interactions with common myeloid progenitors, leading to monocytosis and infiltration, thereby hindering regression of atherosclerosis (58). The feed-forward mechanism of the AGE-RAGE signaling pathway maintains endothelial activation via receptor-mediated internalization of the RAGE ligand and upregulation of RAGE (27, 59), thereby explaining the sustained endothelial phenotype observed in T2DM. The oxidative environment generated by AGE-RAGE signaling further impairs PTP-dependent regulation of adhesion molecule downstream signaling; redox-mediated PTP inactivation has been shown to alter integrin- and receptor tyrosine kinase-dependent migration responses in a temporally and spatially restricted manner, with pathological consequences in metabolic and cardiovascular disease (60). Consistently, hyperglycemia-induced ROS accumulation has been shown to directly impair VEGFR-2-dependent endothelial migration responses in HUVECs, establishing a mechanistic link between the metabolic oxidative milieu and endothelial functional defects (61).

Apart from the adhesion/TEM dissociation, the increased abluminal residence time on hyperglycemia-treated endothelium (**Figure 4A**) and T2DM serum-treated endothelium (**Figure 4B**) was another major finding. TNF-α, however, led to the acceleration of abluminal egress (**Figure 4C**), and survival analysis confirmed progressively slower egress kinetics from TNF-α through control to hyperglycemic conditions (**Figure 4D**). This phenotype also correlates with the previously described reverse transendothelial migration (rTEM) impairment, as previously reported in our earlier study (10), wherein T2DM conditions increased JAM-3 MAC-1 integrin adhesion, leading to the impairment of reverse transendothelial migration. This study confirms endothelial JAM3 upregulation via qPCR specifically under T2DM conditions (**Figure 7C**) and not under TNF-α (**Figure 7D**).

JAM-C was initially identified as a gatekeeper of unidirectional monocyte transmigration, and subsequent studies revealed that divergent JAM-C expression in atherosclerotic plaques accelerates the egress of monocyte-derived cells, linking JAM-C regulation to plaque progression and regression (11, 62). The paradox with the upregulation of JAM3 with T2DM conditions, along with the downregulation of the other junctional adhesion molecules, results in a harmful environment wherein the migrating monocyte traverses through the disrupted endothelial junction, only to be trapped abluminally via the JAM3 MAC-1 integrin adhesion.

This retention-based mechanism can explain the paradox of atherosclerosis in T2DM, characterized by defective monocyte recruitment. Llodrá et al. found that a higher monocyte efflux from plaques is associated with plaque reduction, and a lower monocyte efflux is associated with plaque progression (45). LXR-mediated CCR7 upregulation results in optimal monocyte efflux during plaque regression (63), supporting the balance of recruitment, local proliferation, and retention described by Randolph (64). Our data now expand this model to include the fact that T2DM results in a dual dysfunction in transmigration: a dysfunction in forward transendothelial migration because of disorganized junctions, and a dysfunction in reverse transendothelial migration because of JAM-3/MAC-1 entrapment. Since even limited transmigration can greatly influence plaque composition because of defective efflux, this can explain the basis of the augmented subendothelial monocyte pool in diabetic atherosclerosis, as demonstrated by Woollard and Geissmann (65) and Flynn et al. (66). In fact, diabetes has been found to influence macrophage phenotypes in plaque regression and to hinder phenotypic changes from inflammatory to tissue repair phenotypes, even in the presence of lipid-lowering therapies (67). In addition, the retention effect is augmented by the fact that diabetes compromises the interaction of monocytes and vascular smooth muscle cells in hyperglycemic environments, resulting in subendothelial differentiation and foam cell formation (68, 69).

These findings have significant therapeutic implications. First, since T2DM and TNF-α activate the endothelium through separate programs, anti-inflammatory therapies aimed at NF-κB or TNF-α may not be sufficient to counteract metabolic stress mediated by AGE-RAGE signaling. For example, the CANTOS study showed that the IL-1β-blocking antibody canakinumab reduced recurrent cardiovascular events in hsCRP-positive patients, although the AGE-RAGE axis was not affected (70, 71). These findings highlight the potential benefits of RAGE signaling-targeting therapies, including sRAGE or inhibitors of AGE formation, in the treatment of the diabetic endothelial phenotype (57, 59). Second, JAM-3-mediated monocyte retention highlights restoration of reverse transendothelial migration as a valid therapeutic strategy. Anti-JAM-C antibodies inhibited xenografted tumor engraftment in models of mantle cell lymphoma by disrupting JAM-B/JAM-C interactions (72). Such a strategy may be applied to facilitate monocyte egress and thereby enhance plaque regression, as has been achieved in aortic arch transplant models (11). Additional data suggest that suppression of monocyte recruitment can be sufficient to mobilize macrophages in regression (73, 74). Third, the VCAM1-biased adhesion pattern suggests that suppression of all adhesion may not be necessary to facilitate monocyte transmigration, and balanced expression of adhesion molecules may be more important for efficient adhesion and transmigration.

Several limitations should be acknowledged. Flow assays used human umbilical vein endothelial cells (HUVECs), which may not fully recapitulate the stimulus-specific responses of organ-specific endothelial populations; for example, human coronary artery endothelial cells (HCAECs) exhibit distinct functional responses to vascular risk factor stimuli, including differential regulation of VEGFR-2 and cytoskeletal remodeling, which differ from umbilical vein-derived cells (75). Though RNA-seq studies used different types of endothelial cells for T2DM (dermal EC) and TNF-α (coronary artery EC), the results obtained with the HUVEC model for the validation with qPCR provide some compensation for these limitations. Serum from individuals with T2DM contains many bioactive compounds apart from AGEs, and the role of the other components needs to be worked out. A further limitation of the current study is that the phenotypic identity of monocytes was not assessed following TEM or rTEM. Prior work has shown that monocytes undergoing rTEM *in vivo* acquire a pro-inflammatory CD14^+^CD16^+^ non-classical-like surface phenotype that may contribute to systemic vascular inflammation (11). Whether T2DM serum or hyperglycemic conditions selectively alter the surface expression of CD16, CX3CR1, or JAM-C on transmigrated vs. non-transmigrated monocytes, and whether impaired rTEM under diabetic conditions prevents the phenotypic reprogramming associated with reverse migration, represents an important question for future studies, addressable by post-assay flow cytometry or imaging mass cytometry of recovered cells from the Ibidi µ-Slide VI channel outlet. Whether patient serum from other TNF-driven chronic inflammatory conditions — such as Crohn’s disease or rheumatoid arthritis — recapitulates the retention-based monocyte trafficking phenotype observed under T2DM conditions or instead mirrors the efficient recruitment-and-egress program induced by recombinant TNF-α, represents an important and clinically relevant question that warrants direct experimental comparison in future studies.

Several transcriptional targets with potential relevance to the T2DM–monocyte axis were not included in the current qPCR panel and represent important directions for future investigation. CCL2 (MCP-1), a canonical TNF-α– and NF-κB–regulated chemokine produced by activated endothelium, is a key recruiter of classical CD14^++^CD16^-^ monocytes and its endothelial expression under T2DM serum versus TNF-α stimulation — particularly under flow conditions — warrants dedicated quantification in future studies. DLL1, a Notch ligand expressed on vascular endothelium, participates in the specification and maintenance of non-classical CD14^+^CD16^+^ monocytes through Notch3 signaling in bone marrow progenitors; whether endothelial DLL1 expression is altered by T2DM or hyperglycemic stimuli has not been characterized. CSF1 (M-CSF), the primary survival and differentiation factor for the monocyte–macrophage lineage, is produced by endothelial cells and has been implicated in monocyte retention at atherosclerotic lesion sites. Future studies employing RNA-seq or targeted NanoString profiling of HUVEC monolayers stimulated under physiological flow conditions in the Ibidi µ-Slide VI system would allow simultaneous assessment of CCL2, DLL1, and CSF1 alongside the adhesion molecule and junctional gene panel described here, providing a more complete picture of the endothelial transcriptional response to diabetic stimuli under hemodynamic conditions. Despite these limitations, convergent evidence from functional assays, transcriptomics, GO enrichment analysis, and qPCR and flow cytometric validation provides robust support for the proposed mechanistic framework.

## Data Availability

The datasets presented in this study can be found in online repositories. The names of the repository/repositories and accession number(s) can be found below: https://www.ncbi.nlm.nih.gov/geo/, accession numbers: GSE92724 and GSE134489.

## Supplementary material

Supplementary Figure 1 Schematic of the microfluidic monocyte adhesion assay under physiological flow conditions.

Supplementary Figure 2 Representative time-lapse micrographs illustrating differential monocyte adhesion, transendothelial migration, and reverse transendothelial migration under hyperglycemic versus TNF-α–stimulated conditions.

Supplementary Figure 3 Gene Ontology Biological Process and Cellular Component enrichment analyses reveal orthogonal pathway signatures in T2DM and TNF-α–stimulated endothelial cells.

Supplementary Figure 4 Gene Ontology Molecular Function enrichment and cross-condition GO term correlation analysis demonstrate near-zero concordance between T2DM and TNF-α endothelial programs.

Supplementary Table 1 RT-qPCR primer sequences used in the study.

Supplementary Table 2 Clinical characteristics of the non-T2DM and T2DM individuals used in the study.
