# Supplementary material for: Flow-based imaging reveals dissociated monocyte adhesion and transmigration patterns between TNF-α and diabetes-induced vascular inflammation
Source: Front Immunol. 2026 Jul 10;17:1827607. doi: 10.3389/fimmu.2026.1827607 (PMC13403075; doi:10.3389/fimmu.2026.1827607)
Supplement: Supplementary Figure 1 — Schematic of the microfluidic monocyte adhesion assay under physiological flow conditions. [file DataSheet1.pdf]

Supplementary Material

1 Supplementary Tables

1.1 Supplementary Table 1

RT-qPCR primer sequences used in the study

Gene	Forward primer sequence (5' -3')	Reverse primer sequence (5' -3')
**hICAM1**	GCTTCGTGTCCTGTATGGC	CTGGCGTTATAGAGGTACG
**hVCAM1**	TTCTGAGAGTGTCAAAGAAGG	AAGGAGGATGCAAAATAGAG C
**hE-Selectin**	GCACTGTGTGCAAGTTCGC	GGCTTTTGGTAGCTTCCGTC
**hP-Selectin**	TTCCTGTAACACAAGCCACA	TTGTGAGTCAGATGCTCCAG
**hJAM1**	CAGCTCCTGTGGGGAAAG	TCCGATTGGTGTAAGGAACC
**hJAM-2**	CTGCGCTACCTGGTGGTC	CTACTGCTGTGACTACTTGTTG GTC
**hJAM3**	AAGGACGACTCTGGGCAGT	CCAATAATTCCGCCAATGTT
**hRAGE**	TGGAACCGTAACCCTGACCT	CGATGATGCTGATGCTGACA
**h NF-κB p65 (RELA)**	ATGGCTTCTATGAGGCTGAG	CACAGCATTCAGGTCGTAGT
**hCX3CL1**	AGCTTGCCTCAATCCTGCATCC	TCCTTCAGGAACAGCCACCAG T
**hIL8**	GAGAGTGATTGAGAGTGGACCAC	CACAACCCTCTGCACCCAGTT T
**hYWHAZ**	ACTTTTGGTACATTGTGGCTTCAA	CCGCCAGGACAAACCAGTAT

1.2 Supplementary Table 2

Clinical characteristics of the non-T2DM and T2DM individuals used in the study

	Non-DM	T2 DM	significance
N	12	12	
Age (years)	56±17.8	63±7.2	n.s.
Sex (male/female)	9/3	8/4	n.s.
BMI (kg/m^2)	26.5±3.2	36.9±4.9	<0.001
HbA1c (%)	5.33±0.25	8.64±1.02	<0.001
Glucose (mmol/L)	5.06±0.44	12.7±4.75	<0.001
Smoking (yes/no)	1/11	1/11	n.s.
Hypercholesterolaemia (yes/no)	6/6	0/12	0.006

2 Supplementary Figures

2.1 Supplementary Figure S1

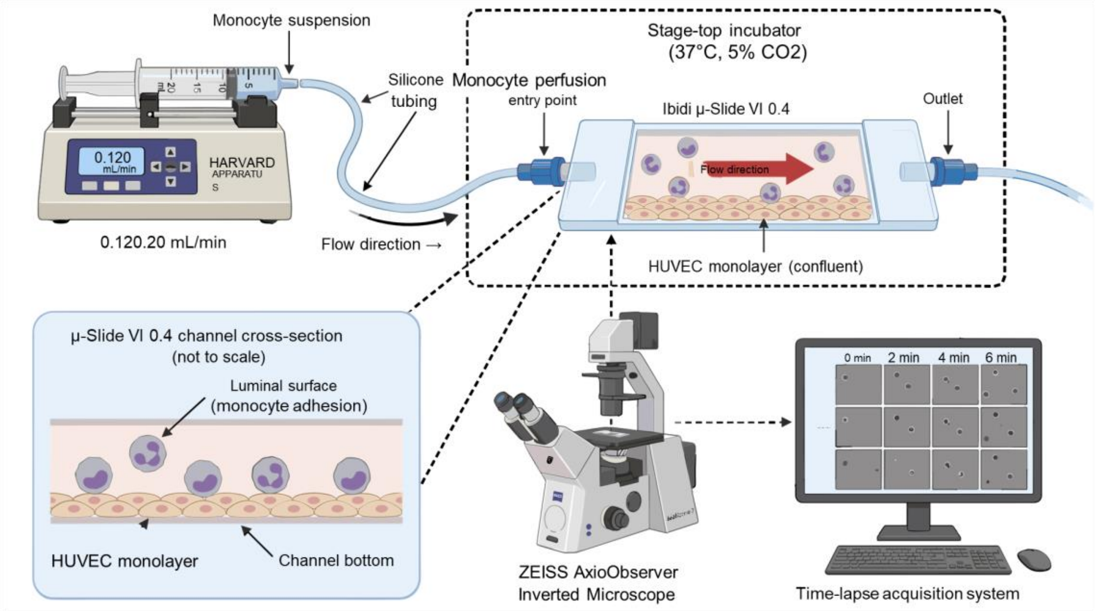

2.2 Supplementary Figure S2

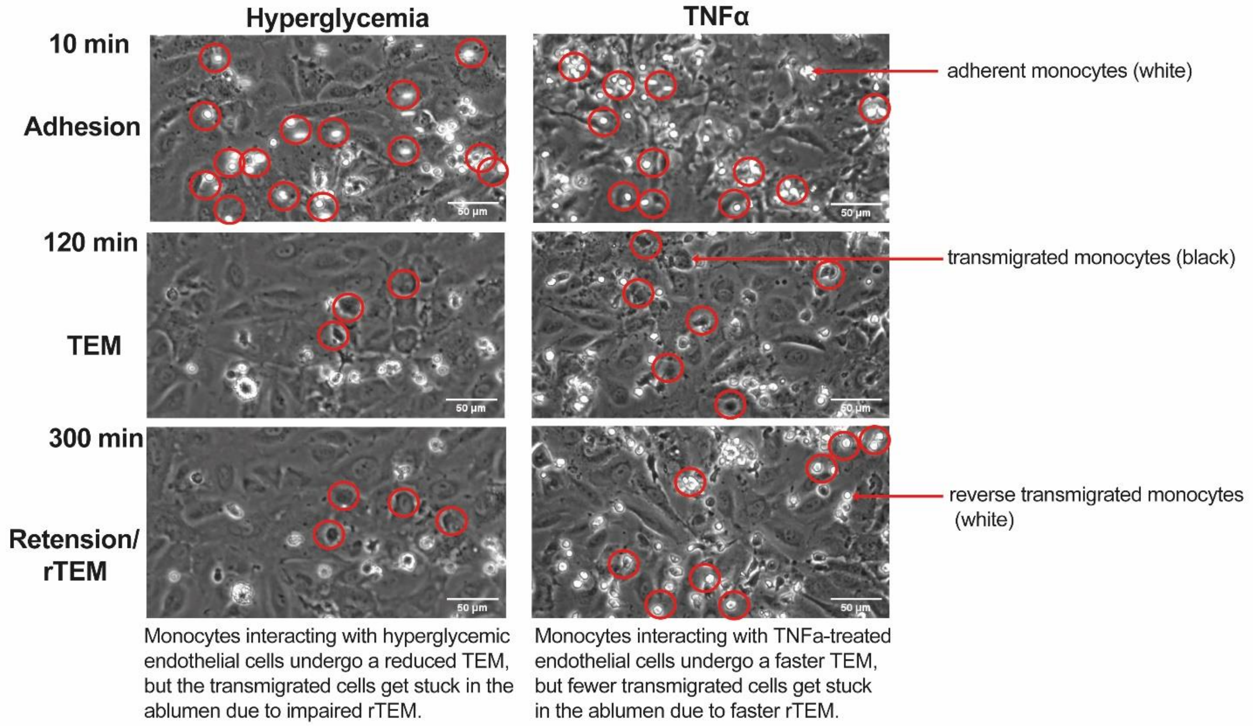

2.3 Supplementary Figure S3

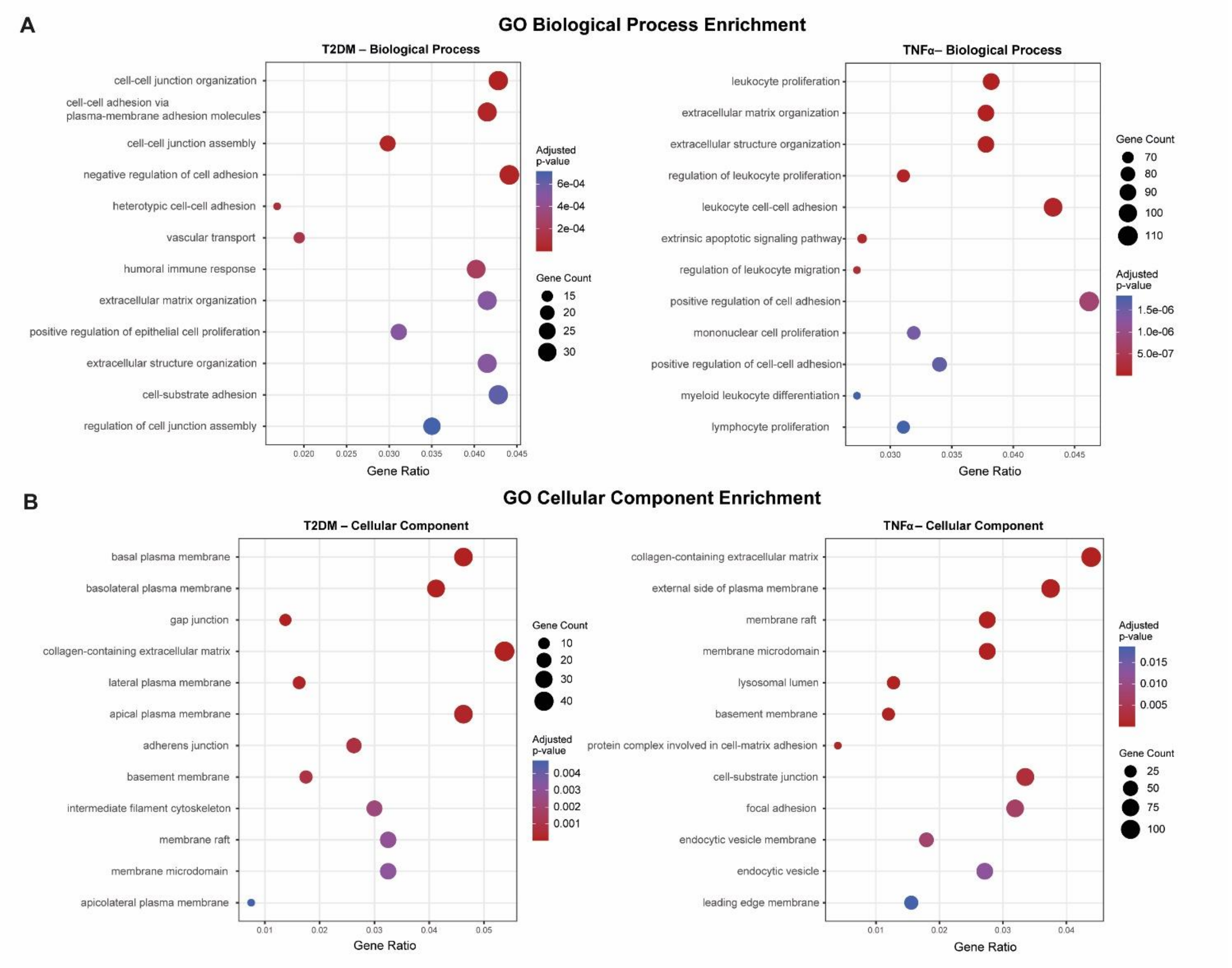

2.4 Supplementary Figure S4

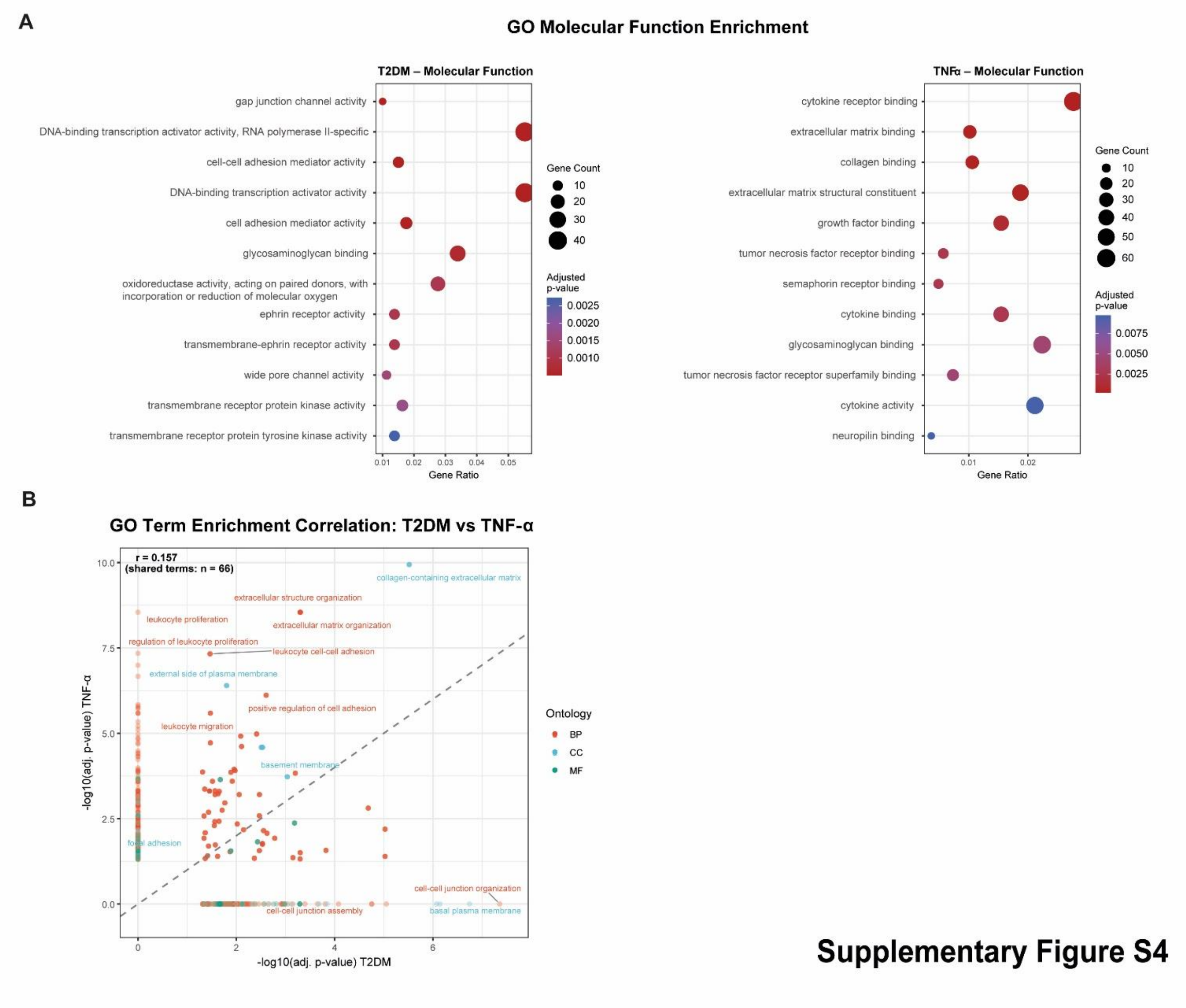

Supplementary Figure S4

Supplementary Figure legends

Supplementary Figure S1. Schematic of the microfluidic monocyte adhesion assay under physiological flow conditions.

Human monocytes were perfused over a confluent HUVEC monolayer cultured in an Ibidi μ-Slide VI 0.4 flow chamber maintained at 37°C and 5% CO_2 in a stage-top incubator. A syringe pump delivered monocyte suspension at a controlled flow rate of 0.12 mL/min through silicone tubing connected to the inlet port of the μ-Slide, generating laminar flow across the endothelial monolayer in the direction indicated. The cross-sectional inset illustrates monocyte interaction at the luminal surface of the HUVEC monolayer, with cells settling and adhering at the apical endothelial surface under flow. Monocyte adhesion events were recorded in real time using a ZEISS AxioObserver inverted microscope equipped with a time-lapse acquisition system, capturing sequential frames at 2-minute intervals to quantify the interactions of monocytes (adhesion, TEM and rTEM) with the endothelial cells per field of view.

Supplementary Figure S2. Representative time-lapse micrographs illustrating differential monocyte adhesion, transendothelial migration, and reverse transendothelial migration under hyperglycaemic versus TNF-α–stimulated conditions.

Phase-contrast images were acquired at 10, 120, and 300 minutes following monocyte perfusion over HUVEC monolayers pre-conditioned with hyperglycaemia (left column) or TNF-α (right column) under physiological flow. Red circles denote monocytes at each stage of the interaction cascade. At 10 minutes (Adhesion), surface-adherent monocytes appear as bright white refractile bodies on the luminal endothelial surface. At 120 minutes (TEM), transmigrated monocytes are identified by their dark, phase-dim morphology beneath the endothelial monolayer. At 300 minutes (Retention/rTEM),

monocytes that have undergone reverse transendothelial migration (rTEM) reappear as white refractile cells at the luminal surface. Monocytes interacting with hyperglycaemic endothelial cells display reduced TEM but accumulate in the abluminal compartment due to impaired rTEM. In contrast, monocytes interacting with TNF-α–stimulated endothelial cells undergo faster and more efficient TEM, with fewer cells retained abluminally owing to accelerated rTEM. Scale bars = 50 μm.

Supplementary Figure S3. Gene Ontology Biological Process and Cellular Component enrichment analyses reveal orthogonal pathway signatures in T2DM and TNF-α–stimulated endothelial cells.

GO enrichment analyses were performed on differentially expressed genes from T2DM (GSE92724) and TNF-α–stimulated (GSE134489) endothelial cells using clusterProfiler (v4.16.0; BH-adjusted $p < 0.05$). Dot plots display the top enriched terms ranked by gene ratio; dot size reflects gene count and colour intensity reflects statistical significance (blue: less significant; red: most significant). (A) GO Biological Process enrichment. T2DM endothelial cells are enriched for junctional and adhesive programmes including cell-cell junction organisation, cell-cell junction assembly, cell-substrate adhesion, and negative regulation of cell adhesion, consistent with structural endothelial remodelling. TNF-α–stimulated endothelial cells display enrichment for leukocyte proliferation, leukocyte cell-cell adhesion, positive regulation of cell adhesion, and myeloid leukocyte differentiation, consistent with a pro-inflammatory activation programme. (B) GO Cellular Component enrichment. T2DM endothelial cells show enrichment for basal and basolateral plasma membrane compartments, collagen-containing extracellular matrix, basement membrane, adherens junction, and membrane raft terms, reflecting chronic basement membrane remodelling and altered junctional architecture. TNF-α–stimulated endothelial cells are enriched for the external side of the plasma membrane, membrane raft, membrane microdomain, focal adhesion, and cell-substrate junction compartments, consistent with surface display of adhesion molecules that facilitate leukocyte capture.

Supplementary Figure S4. Gene Ontology Molecular Function enrichment and cross-condition GO term correlation analysis demonstrate near-zero concordance between T2DM and TNF-α endothelial programmes.

(A) GO Molecular Function enrichment (BH-adjusted $p < 0.05$; clusterProfiler v4.16.0). T2DM endothelial cells show enrichment for gap junction channel activity, cell-cell adhesion mediator

activity, glycosaminoglycan binding, DNA-binding transcription activator activity, and transmembrane receptor protein kinase activity, reflecting altered junctional communication and ECM-receptor signalling under metabolic stress. TNF-α–stimulated endothelial cells display enrichment for cytokine receptor binding, cytokine binding, tumor necrosis factor receptor binding, TNF receptor superfamily binding, and cytokine activity, consistent with coordinate cytokine-driven inflammatory signalling. (B) Cross-condition GO term correlation scatter plot. Each point represents a GO term shared between the T2DM and TNF-α enrichment results across all three ontology domains (BP, orange; CC, salmon; MF, teal; $n = 66$ terms), plotted as −log10(BH-adjusted p-value) for T2DM (x-axis) versus TNF-α (y-axis). The dashed diagonal indicates perfect concordance. The Pearson correlation coefficient ($r = 0.157$) demonstrates near-zero concordance between the two enrichment profiles, confirming that T2DM and TNF-α engage qualitatively non-overlapping molecular programmes at the pathway-ontology level.
